# The Aversive Racism Theory of Cultural Appropriation: Attributions of Target Intent Suppresses Evaluations of Intergroup Harm

**DOI:** 10.1177/01461672241292427

**Published:** 2024-12-13

**Authors:** Ariel J. Mosley

**Affiliations:** 1 University of California, Davis, USA

**Keywords:** cultural appropriation, intergroup harm, social cognition, intent, collective action

## Abstract

This research investigates whether racially dominant (White) and minoritized group members (Black) differentially evaluate intergroup harm in ambiguous (vs. overt) acts of cultural appropriation (the aversive racism hypothesis), due to attributions of positive intentions to the target (the intent as justification hypothesis). Four experiments (*N* = 1,020, 3 preregistered) and an internal meta-analysis converge to demonstrate that White perceivers evaluated less harm than Black perceivers in ambiguous acts of cultural appropriation. Attributions of positive intent served as a mechanism underlying this effect; naturally occurring variations in positive intent mediated the link between participant race and harm evaluations (Studies 2 and 3), and experimentally manipulating target intent altered harm evaluations as well as motivations for collective action (Study 4). Findings integrate work from multiple academic disciplines with insights from contemporary theories of prejudice to suggest that perceivers’ attributions of positive intent can obscure their evaluations of harm in acts of cultural appropriation.

When do people’s assumptions of an actor’s positive intent suppress their evaluations of harm in acts of cultural appropriation? Cultural appropriation has been defined as “the taking—from a culture that is not one’s own—of intellectual property, cultural expressions or artifacts, history and ways of knowledge” (Ziff & Rao, 1997, p. 1). As there has been increased media attention devoted to racial justice movements (i.e., Black Lives Matter; Leach & Allen, 2017), there has also been an increased societal emphasis on values of egalitarianism (Charlesworth & Banaji, 2019), as well as prevalent beliefs, particularly among dominant group members, that racial discrimination is a thing of the past (Horowitz et al., 2019). Despite these intentional efforts for positive social change, racial tensions have become increasingly hostile and complex across the globe (Bhattacharyya et al., 2016; Winant, 2020), leaving many to wonder how to successfully navigate sensitive intergroup topics such as cultural appropriation (Page-Gould et al., 2022). Indeed, groups disagree about the extent of harm that takes place as a result of culturally appropriative actions (Mosley & Biernat, 2021).

While cultural appropriation is a topic that has been frequently discussed in broader social sciences (e.g., sociology, media and communication studies) and humanities (e.g., critical race studies, law, philosophy), it has been a burgeoning topic in the psychological literature. Synthesizing key insights from these interdisciplinary perspectives on cultural appropriation with theories in multiple areas of social psychology (e.g., moral and social cognition, intergroup relations, cultural psychology), the current research investigates people’s evaluation of harm in culturally appropriative acts, and whether people’s assumptions about an actor’s motivations can suppress their evaluations of harm. Specifically, the current research investigates the following: (H1) The *aversive racism theory of cultural appropriation*: White perceivers will evaluate less harm than Black perceivers in ambiguous, but not overt, acts of cultural appropriation; and (H2) *The intent as justification hypothesis*: attributions of positive actor intent will explain group differences in evaluations of harm.

## On the Perceived Harm of Cultural Appropriation

A group’s culture plays a fundamental role in how we make sense of ourselves and our social environments (Allport, 1954; Bruner, 1957), and fulfills an important self-categorization function for group members by allowing them to delineate group boundaries, social norms, and valued ways of being in the world (Dragojevic & Giles, 2016; Keblusek et al., 2017; Turner et al., 1987). Culture also enriches people’s psychological lives by providing them a sense of identity continuity, existential value, and collective meaning (Kashima, 2010; Solomon et al., 2004). For groups who have been historically subjected to racial oppression, the practice and expression of one’s culture can provide them freedom and autonomy to transcend *beyond* their marginalized status in society through the creation of counternarratives about the group that allow for distinct culturally anchored meanings and life stories (Etoke, 2022; Jackson, 2020; McAdams, 2001). Thus, appropriating cultural products from subordinated groups can be experienced as particularly traumatic and psychologically disruptive because it can threaten the group’s ability to maintain their collective sense of existential value and continuity (Etoke, 2022; Jackson, 2020; Kashima, 2010; Lenard & Balint, 2020).

In investigating the ethics of cultural appropriation, philosopher Young (2005, 2010) locates the impact of the act (and its culpability for moral offensiveness) in terms of the target’s individual intent. However, this perspective may discount the sociocultural significance of racial hierarchies and group privilege (Blumer, 1958; Bobo, 1988; Bonilla-Silva, 2006; Feagin, 2020).

An alternative way that scholars have typified the construct of cultural appropriation is by examining the sociopolitical locations, and *differential power* (i.e., privileged access to status and resources), between the target engaged in the act and the source community from whom the cultural product originates (Rogers, 2006). Mosley and Biernat’s (2021) *perpetrator prototypicality model of cultural appropriation* asserts that dominant (e.g., White Americans) and minoritized (e.g., Black Americans) groups disagree on the extent to which they label acts of outgroup cultural use as culturally appropriative, because cultural appropriation signals threats to ingroup distinctiveness for low-status group members. Across five experiments, Black perceivers were more likely to label scenarios of White actors taking from Black culture (e.g., a White-owned company selling dreadlocked hair, a White artist performing rap music) as appropriative. White perceivers did not differentiate between White actors engaged in Black culture and Black actors engaged in “White culture,” suggesting a power-neutral way of making judgments of cultural appropriation.

More recent work provides additional evidence demonstrating the importance of sociopolitical location and historical context in understanding people’s perceptions of cultural appropriation. Mosley, Biernat, and Adams (2023) showed *when* people are most likely to make power-neutral judgments about acts of cultural appropriation (e.g., equating the actions of White actors and Black actor) by investigating the role of *colorblind racism*, or a denial of racial inequalities and realities (Bonilla-Silva, 2006). Black *and* White individuals high in colorblind racism were most likely to evaluate scenarios of cultural exploitation and scenarios of cultural dominance as equally appropriative, justified, and racist. Such findings have important implications for understanding how judgments of cultural appropriation are malleable based on the racial ideologies, knowledge, and motivations of the perceiver. Indeed, group positioning, power, and status play crucial roles in how people define acts of cultural appropriation, but what about people’s evaluations of intergroup harm?

While previous work in psychology has primarily explored the status of the perceiver and the status of the actor as it relates to perceptions of cultural appropriation (Finkelstein & Rios, 2022; Kirby et al., 2023; Mosley & Biernat, 2021; Mosley, Biernat, & Adams, 2023), more recent research has begun to explore the role of perceived harm that it can have for the source community. To the extent individuals identify greater *harm* in acts of outgroup cultural use, they are more likely to identify such acts as “cultural appropriation” (Mosley, Heiphetz, et al., 2023). Similarly, in a qualitative study interviewing Black Americans, Twali and colleagues (2023) revealed cultural appropriation as a pervasive theme in their everyday lives that harms their sense of collective power and ability to influence others, such as when music record label companies lack Black ownership, despite the prolific influence of Black American culture. Despite these potential harms enacted toward the source community, no work has explored why group members disagree about the *harm* implicated from cultural appropriation.

## The Aversive Racism Theory of Cultural Appropriation

One key factor that may influence how people reason about the harm implicated in acts of cultural appropriation may be the perceived motivations of the actor involved, most notably attributions of *positive* intention. By intention, this article refers to the deliberate and conscious wishes of people who inhabit and animate particular realities and ways of being (Kurtiş et al., 2010). Certainly, perpetrator *intent* is often a necessary factor for perceived culpability in evaluations of actions as just, moral and fair (Alicke et al., 2015; Cushman, 2008; Folger & Cropanzano, 2001), legal decisions (Dripps, 2003; see also Ames & Fiske, 2015), as well as in evaluation of prejudice and discrimination (Swim et al., 2003). However, we know from psychology that intended attitudes and actual outcomes do not always align (Dovidio et al., 2002; Greenwald & Banaji, 1995), and that people who have positive intentions to honor or commemorate a particular group can inadvertently cause harm to that group, regardless of their conscious individual intentions (Ahmed, 2004; Kurtiş et al., 2010; Salter & Adams, 2016).

Classic theories of prejudice emphasize how a focus on the intent of the actor obscures the conditions and social relations that perpetuate conditions of intergroup oppression (Allport, 1954; Blumer, 1958; Crandall & Eshleman, 2003; Dovidio et al., 2002). Similarly, racialized discrepancies of perceived harm from acts of cultural appropriation may be most likely to occur when the intent of the actor is *ambiguous* and motivations can be explained away (Gaertner & Dovidio, 1986, 2000). Indeed, dominant group members (e.g., White Americans) may be more lenient compared with minoritized group members (e.g., Black Americans) when making harm evaluations of ambiguous cultural appropriation, where the intent of the actor can be justified as positive. For many Black perceivers—who are positioned in a sociopolitical space where they are more likely to experience the harms of cultural appropriation against a backdrop of individual, cultural, and structural racism (Eberhardt, 2019; Roberts & Rizzo, 2021)—attributions of actor intent may be less of a factor when evaluating intergroup harm. In contrast, positive intent may not serve as an adequate justification in overt acts of cultural appropriation, as in cases of Blackface, cultural parties, or minstrel shows that are normatively associated with racist and anti-Black attitudes (Lott, 1995).

On this note, contemporary theories of group-based oppression and discrimination also suggest that oppression often manifests in subtle forms and is expressed in ambiguously framed situations where behavioral expectancies and social norms are unclear (Gaertner & Dovidio, 1986). Pettigrew and Meertens (1995) differentiate between “blatant” and “subtle” natures of racism. Subtle racism occurs in the context of everyday routines and everyday social interactions, where it is not clear whether the behaviors or intentions are prejudicial. Such actions can be easily rationalized or interpreted as irrelevant to race. This is in contrast to “old-fashioned” forms of racism that are more recognizable, involves blatant antipathy, and is expressed in more direct, but less socially acceptable ways (McConahay, 1986).

Relative to overt discrimination, subtle discrimination can involve an unclear intent to cause harm to a target, is more difficult to detect, and is often categorized as low in intensity (Jones et al., 2016). As subtle discrimination is more difficult to identify, it can be particularly harmful because targets are more likely to ruminate over whether it occurred in the first place (Farber et al., 2021), and more often make individual attributions and blame themselves for prejudicial outcomes (Hebl et al., 2002; King et al., 2011), which can cause more psychological distress than explicit discriminatory actions (Crocker et al., 1991; Salvatore & Shelton, 2007; Singletary & Hebl, 2009). Moreover, subtle forms of discrimination are more pervasive and occur more frequently than overt forms (Utsey et al., 2002; Utsey & Ponterotto, 1999; Van Laer & Janssens, 2011), causing more chronic stress and greater depressive symptomatology (Crouter et al, 2006; McGonagle & Kessler, 1990), reduced quality of life (Utsey et al., 2002), and more negative health outcomes such as increased cardiovascular problems (Guyll et al., 2001; Thompson, 1999).

Relative to overt forms of discrimination, subtle discrimination creates greater hesitation and ambivalence for perceivers to label such behaviors as racist, as well as the extent to which such acts are viewed as socially acceptable (Jones et al., 2016), and as a result are justified as trivial, harmless, and unintentional (Hebl et al., 2002; King et al., 2011). Identifying ambiguous forms of discrimination may be particularly difficult to detect because it can require more cognitive and emotional resources to decipher ambiguous discriminatory cues (Crocker & Major, 1989; Salvatore & Shelton, 2007; Singletary & Hebl, 2009). Moreover, scholars have discussed how subtle forms of discrimination are more *recent* phenomena that is a manifestation of contemporary forms of racism (Dipboye & Colella, 2005; Dovidio et al., 1998; Virtanen & Huddy, 1998), whereas overt forms are more clearly understood and recognized in social discourse and in the scientific literature (Dovidio et al., 2002; Jones et al., 2016). Thus, it is critical to experimentally examine how cultural appropriation can occur as a contemporary form of racism that manifests in both overt and ambiguous forms.

In the same way, acts of cultural appropriation can also range from hostile and overtly discriminatory, to more ambiguous and seemingly innocuous (Mosley & Biernat, 2021; Young, 2005). The use of cultural objects by nonmembers of the group can be blatantly hostile and discriminatory, where the targets’ behaviors and intentions are more clearly prejudicial based on the current social norms (e.g., the use of blackface, the participation in cultural parties). Acts of cultural appropriation can evoke legacies of chattel slavery and colonialism, where Black Americans were considered legal property of White Americans (Harris, 1993; Tarlow, 2024), and can implicate significant racial dehumanization and fetishization (Keefer et al., 2017; Mosley, Bharj, & Biernat, 2023). As a consequence, minoritized cultural products may also be viewed as “up for grabs” and thus actors who engage in cultural appropriation are not bound to the norms of collective ownership (Martinović & Verkuyten, 2024).

On the other end of the continuum, actors’ behaviors and intentions may be more ambiguous (e.g., the commercialization of soul food, the wearing of dreadlocks), complicating the nature of cultural appropriation. Situational ambiguity can provide for a “cover” in which harmful behavior can be mistaken for a neutral or socially acceptable action (Crandall & Eshleman, 2003), where a seemingly positive intentioned act of cultural appropriation can justify potential negative implications of harm (see Thai et al., 2019). Indeed, perceivers view ostensibly well-intentioned acts of intergroup curiosity (such as asking a random Black person whether it is acceptable to dress as a character of a different race for Halloween) as low effort, and thus has negative implications for perceived moral culpability (Mosley & Solomon, 2023). Such actions can prioritize the experience of dominant group members over the potential harm, tokenism, and onus of responsibility that is placed upon on minoritized individuals (Starck et al., 2024; Torrez et al., 2024). Moreover, acts of cultural appropriation are more likely to be interpreted by White perceivers as *cultural celebration* (Mosley & Biernat, 2021). However, positive motivations to celebrate a minoritized group can actually backfire and cause intergroup harm (Thai et al., 2016; Wallace, Reeves, & Spencer, 2024). Indeed, expressing positive stereotypes about minoritized groups that convey subjectively favorable beliefs about social groups can have adverse implications for intergroup relations and perpetuate power inequalities (Cheryan & Bodenhausen, 2000; Czopp, 2008; Czopp et al., 2015).

While such overt and blatant acts of cultural appropriation may be less pervasive in today’s society, aversive racism theory (Gaertner & Dovidio, 1986, 2000) suggests that the nature of racism has changed to become more subtle and ambiguous. Indeed, some ambiguous acts of cultural appropriation can be particularly insidious because they can perpetuate oppressive socially constructed narratives about marginalized group members (Sue et al., 2007). Similar to the racial trauma caused by subtle microaggressions, acts of cultural appropriation can implicitly communicate subordinated positionality to the source community, regardless of the actor’s conscious awareness and intentions (Liu et al., 2019, 2023). For example, in their examination of gendered microaggressions, Lewis and Neville (2015) found that Black women frequently experience interacting with people who “imitated the way they think Black women speak” (p. 295), where they were mocked for what others perceive as a gender-specific form of African American vernacular English (AAVE) or use of culturally appropriative language (e.g., “hey g-i-r-l-f-r-i-e-n-d,” p. 291).

Historically, actions of colonial appropriation have been justified through arguments that such actions were not harmful, but instead had benevolent and had romanticized implications due to positive intentions to help or celebrate oppressed groups (Buescher & Ono, 1996; Kurtiş et al., 2010). In everyday social discourse and in some scholarly debates about cultural appropriation, justifications that an actor has positive intention is a common defense some use to disarm accusations of cultural appropriation (Young 2005, 2010), yet no work has experimentally explored this phenomenon. Indeed, there is much social discourse and experimental work suggesting that dominant group members often respond to acts of cultural exploitation positively (Malik, 2017; Mosley & Biernat, 2021). Moreover, the current research investigates how *prioritizing actor motivations* are dependent on the sociopolitical position of the participant, where dominant group members (e.g., White Americans) may be more likely than minoritized group members (e.g., Black Americans) to attribute *positive intent to the actor* when making judgments about the implications of cultural appropriation.

The current research extends the psychological literature on cultural appropriation in four important ways. First, this work is the first to experimentally examine the roles of situational ambiguity and attributions of actor intent in the context of cultural appropriation on perceptions of intergroup harm as a result of such actions. Second, while previous work examines cognitive categorizations and perceptions (whether a particular behavior or act of intergroup cultural use fits into the prototype of cultural appropriation), this work investigates the *psychological justification processes* that individuals engage in to understand the implications of a cultural appropriation by focusing on the future appraisals (i.e., the extent to which there is potential for intergroup harm). By doing so, this work answers the critical question of *when* minoritized source communities may or may not be protected from harm that results from the direct appropriation of their culture and resources. Third, this work investigates evaluations of intergroup harm by considering the assumed psychology of the target as a critical moderator, determining how perceived positive, negative, and neutral intent changes people’s evaluations of intergroup harm. Finally, this work investigates the downstream implications of perceiver status and target intent on collective action to fight anti-Black discrimination.

## The Current Research

Specifically, the present research investigates group members’ perceptions of intergroup harm as it relates to perceived target intent in ambiguous and overt scenarios of *cultural exploitation* (high-status targets engaging with aspects of a subordinated culture). In Study 1, Black and White participants evaluated implications of harm in ambiguous cultural appropriation and overt cultural appropriation. Studies 2 and 3 examine whether attributions of intent serve as an underlying mechanism explaining intergroup differences in harm evaluations. Study 4 investigates the causal role of intent in people’s evaluations of harm as well as downstream consequences on collective action. An internal meta-analysis investigates the effect across studies.

## Study 1

Study 1 provided an initial test of whether Black and White participants differentially evaluate harm enacted toward the source community as a result of ambiguous and overt acts of cultural appropriation. Participants read about White targets who (a) engaged in an ambiguous act of cultural appropriation (i.e., a White author writing about the subjective experience of Black people) and (b) engaged in an overt act of cultural appropriation (i.e., a White fraternity having a “cultural party” performing the subjective experience of Black people). Scenarios were chosen based on the type of cultural object to represent *subjective cultural appropriation* (e.g., outgroup cultural use involving the narration of a source communities’ cultural experiences/outgroup cultural use involving the performance of a source communities’ cultural experiences and subjectivity; Young, 2005, 2010).

### Method

#### Participants

The sample size for this study was based on an a priori power analysis using G*Power software (Faul et al., 2007) that conservatively assumed a small effect based on prior research on differences in cognition across perceiver status (e.g., dominant vs. minoritized) and perceptions of cultural appropriation (e.g., Finkelstein & Rios, 2023; Mosley & Biernat, 2021; Mosley, Biernat, & Adams, 2023). A power analysis that estimated a small effect size (*f* = 0.15), a large correlation among repeated measures (0.65), and included other standard parameters (α = .05, and 80% power) for a 2 × 2 mixed (within-between interaction) subjects’ analysis estimated a desired sample size of 92 participants. I oversampled by 10% (10) to account for participant exclusions. Therefore, I aimed to collect a sample size of 104 participants (to be divisible by 4).

A total of 108 adults living in the United States were recruited via Prolific.com. A pre-screening survey was used to recruit Black and White American participants. Five respondents were removed for self-identifying as members of a different racial group during the demographics portion of the study. Three participants were recruited, but did not complete the study, thus were excluded. One participant was removed for requesting to have their data excluded, resulting in a final analytic sample of 99 participants. Based on a sensitivity power analysis in G*Power, this sample size provided 0.80 power to detect an interaction effect of Participant Race × Appropriation Ambiguity of 0.17 or greater.

The 99 participants whose responses were included in analyses ranged from 20 to 72 years old (*M* = 40.78 years, *SD* = 13 years). Participants self-identified their race as White American (51.5%) or Black American (48.5%) and their gender as female (47.5%), male (51.5%), and <1% self-identified as “other gender.” All materials and procedures described below and in subsequent studies were approved by the authors’ affiliated university institutional review board (IRB). In this and all subsequent studies, all measures, manipulations, and exclusions are reported. Upon publication, the data will be posted to the following repository (https://osf.io/smrfu/?view_only=2b9a54f7e1c34c2b8b08613bdff48544).

#### Design and Procedures

This study adopted a 2 (Participant Race: White vs. Black—Between) × 2 (Appropriation Ambiguity: Ambiguous vs. Overt—Within) mixed design. Participants learned that the purpose of the study was to “understand what people think about different social situations.” Modeled after previous research (Mosley & Biernat, 2021; Mosley, Biernat, & Adams, 2023; Mosley, Heiphetz, et al., 2023), participants read a standard definition of cultural appropriation (Rogers, 2006; Scafidi, 2005; Ziff & Rao, 1997) prior to their evaluations: “Cultural appropriation refers to taking on elements of a culture other than one’s own. This can involve taking or using intellectual property, traditional knowledge, cultural expressions, or artifacts from someone else’s culture without permission.”

Participants read and evaluated descriptions of a possible case of cultural appropriation. Scenarios were directly taken from previous research manipulating cultural exploitation (Mosley & Biernat, 2021; Mosley, Biernat, & Adams, 2023) that were adapted from actual news and social media articles that labeled incidents of cultural appropriation—where a target engages with a cultural product that is not representative of their racial group. These scenarios introduced the appropriation ambiguity manipulation.

In a counterbalanced order, participants read about both (a) an *ambiguous* case of cultural appropriation and (b) an *overt* case of cultural appropriation. The two conditions differed with respect to how culturally appropriative they were perceived to be in previous research, but were similar in terms of the ontological nature and the *type of appropriation* that occurred. Both scenarios reflected *subject appropriation*, where a target appropriates an outgroup epistemic object such as an intellectual idea, a way of being in the world, or a subjective cultural narrative experience (Young, 2005, 2010). In this case, a high-status target (e.g., White American actor) engaged in the imitation/simulation of the subjective experience of a low-status (e.g., Black Americans) source community. Moreover, the vignettes were similar with respect to word count and were analogous to other research that investigated acts of cultural appropriation using a more diverse set of scenarios (Mosley, Biernat & Adams, 2023).

The “Ambiguous Appropriation Condition” described a scenario about a White author writing a novel narrating from the subjective experiences and perspectives of Black people.

Specifically, the ambiguous appropriation condition was chosen to reflect outgroup cultural use for which uninvolved bystanders would exhibit more hesitation and would experience more affective and cognitive ambivalence to label the behavior as “cultural appropriation” (Jones et al., 2016), and consistently scored the *lowest* of eight scenarios on perceptions of cultural appropriation in prior work (*M*_perceived appropriation_ ranged from 3.09 to 3.13 on a 7-point scale; Mosley & Biernat, 2021). Similarly, recent research demonstrates that perceivers tend to rate an analogous scenario as *particularly low* on perceptions of cultural appropriation, “*A White American author writes a book about the lives of African American people*” relative to 56 other scenarios of outgroup cultural use (*M*_perceived appropriation_ ranged from 3.10 to 3.37 on a 7-point scale; Mosley, Heiphetz, et al., 2023; Studies 1–2).

The “Overt Appropriation Condition” described a scenario about a White fraternity throwing a “ghetto-themed” Compton Cookout party where party goers were encouraged to perform the subjective experiences and perspectives of Black people. The overt appropriation condition was chosen to reflect more explicitly negative treatment toward a source community that is less likely to produce hesitation and ambivalence within an uninvolved bystander to label the behavior as “cultural appropriation” (Jones et al., 2016), and consistently scored the *highest* of eight scenarios on perceptions of cultural appropriation in prior work (*M*_perceived appropriation_ ranged from 4.29 to 4.37 on a 7-point scale, Mosley & Biernat, 2021). Moreover, recent research demonstrates that perceivers tend to rate an analogous scenario as *particularly high* on perceptions of cultural appropriation, “A White American fraternity celebrates a ‘Compton Cookout Party’ with stereotypically ‘Black’ music, food, costumes, and dancing” relative to 56 other scenarios of outgroup cultural use (*M*_perceived appropriation_ ranged from 3.48 to 4.93 on a 7-point scale; Mosley, Heiphetz, et al., 2023). Full vignettes used in all studies are available in the supplemental materials.

After reading each scenario, participants indicated their *evaluations of intergroup harm* enacted toward the source community using three items (items adapted from Mosley & Biernat, 2021), such as (a) “[Target]’s actions harm Black culture,” (b) “[Target]’s actions demean Black culture,” and (c) “[Target]’s actions weaken Black culture” (1—*Strongly Disagree*; 4—*Neither Agree nor Disagree*; 7—*Strongly Disagree*). For analyses below, three items were averaged for each condition (Ambiguous: α = .91; Overt: α = .81).

Afterward, participants completed a manipulation check of *perceived overtness* of cultural appropriation (adapted from Mosley, Heiphetz, et al., 2023) using a single item: “This is an overt act of cultural appropriation” (1—*Strongly Disagree*; 4—*Neither Agree nor Disagree*; 7—*Strongly Disagree*). Participants then completed demographics and were debriefed.

### Results

#### Manipulation Check

First, to assess the effectiveness of the manipulation check, perceptions of cultural appropriation was analyzed using a 2 (Participant Race: White vs Black—Between) × 2 (Appropriation Ambiguity: Ambiguous vs. Overt—within) mixed-design analysis of variance (ANOVA). Each effect was as follows: (a) Participant Race, *F* (1,97) = 2.45, *p* = .121, *partial* η^2^ = .025, indicating that Black participants (*M* = 4.72, *SD* = 2.04) responded similarly to perceptions of cultural appropriation, overall, as White participants (*M* = 4.27, *SD* = 1.60); (b) Appropriation Ambiguity, *F*(1,97) = 65.63, *p* < .001, *partial* η^2^ = .404; indicating that all participants evaluated the overt cultural appropriation scenario (i.e., Culture Parties; *M* = 5.41, *SD* = 1.90) as more of an obvious act of cultural appropriation, on average, than the ambiguous scenario of cultural appropriation (i.e., Literature; *M* = 3.56, *SD* = 1.84); (c) Participant Race × Appropriation Ambiguity, *F* (1,97) = 10.43, *p* = .002, *partial* η^2^ = .097.

Simple effects tests based on the interaction indicated that when reading about overt cultural appropriation, White perceivers (*M* = 5.55, *SD* = 1.72) evaluated the scenario as more blatantly appropriative than Black perceivers (*M* = 5.27, *SD* = 2.08), *F* (1,97) = 11.34, *p* < .001, *partial* η^2^ = .11, 95% confidence interval (CI) = [−1.89, −0.49]; throughout the article, the CIs refer to the mean differences for the pairwise comparisons. When reading about ambiguous appropriation, Black perceivers (*M* = 4.17, *SD* = 2.00) evaluated scenarios as similarly blatant as White perceivers (*M* = 2.98, *SD* = 1.48), *F* (1,97) = 0.53, *p* = .47, *partial* η^2^ = .005, 95% CI = [−0.48, 1.04]. In addition, Black perceivers (*M_ambiguous_* = 4.17, *SD_ambiguous_* = 2.00; *M_overt_* = 5.27, *SD_overt_* = 2.08; *F* (1,97) = 11.51, *p* = .001, partial η^2^ = .106, 95% CI = [−0.175, 0.46]) and White perceivers (*M_ambiguous_* = 2.98, *SD_ambiguous_* = 1.48; *M_overt_* = 5.55, *SD_overt_* = 1.72; *F* (1,97) = 66.21, *p* < .001, partial η^2^ = .406; 95% CI = [−3.20, −1.94]) both evaluated ambiguous acts of cultural appropriation as less blatant than overt acts.

#### Perceived Harm

**Figure 1.**
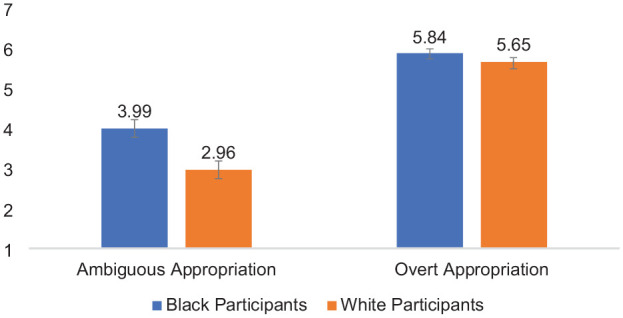
Participant Race × Appropriation Ambiguity Interaction on Evaluations of Harm Toward the Source Community, Study 1. *Note*. Error bars represent standard error bars.

Second, of most relevance to the main question of interest (how Black and White participants evaluate perceived harm for ambiguous vs. overt forms of cultural appropriation), perceived harm was analyzed using a 2 (Participant Race: White vs. Black—Between) × 2 (Appropriation Ambiguity: Ambiguous vs. Overt—Within) mixed-design ANOVA. Each effect was as follows: (a) Participant Race, *F* (1,97) = 5.40, *p* = .022, *partial* η^2^ = .053, indicating that Black participants perceived greater harm, on average, than White participants; (b) Appropriation Ambiguity, *F* (1,97) = 146.20, *p* < .001, *partial* η^2^ = .601; indicating that participants evaluated actors who engaged in overt cultural appropriation as more harmful, on average, than actors who engaged in ambiguous cultural appropriation. Confirming *aversive racism hypothesis of cultural appropriation* (H1), the two-way interaction of Participant Race × Appropriation Ambiguity was significant, *F* (1,97) = 4.95, *p* = .028, *partial* η^2^ = .049, see Figure 1. Means and standard deviations are shown in Table 1.

**Table 1.** Dependent Measures by Participant Race and Appropriation Ambiguity, Study 1.

	Participant race × Appropriation ambiguity			
	Ambiguous appropriation		Overt appropriation	
Measure	White participants	Black participants	White participants	Black participants
	*M (SD)*	*M (SD)*	*M (SD)*	*M (SD)*
Manipulation check	2.98 (1.48)	4.17 (2.00)	5.55 (1.72)	5.27 (2.08)
Evaluations of harm	2.96 (1.43)	3.99 (1.93)	5.65 (1.51)	5.84 (1.51)

Simple effects tests based on the interaction indicated that when reading about overt cultural appropriation, White perceivers (*M* = 5.65, *SD* = 1.51) evaluated the target as similarly harmful than Black perceivers (*M* = 5.84, *SD* = 1.51), *F* (1,97) = 0.41, *p* = .53, partial η^2^ = .004, 95% CI = [−0.80, 0.41]. When reading about ambiguous appropriation, Black perceivers (*M* = 3.99, *SD* = 1.93) reported greater evaluations of harm than White perceivers (*M* = 2.97, *SD* = 1.43), *F* (1,97) = 9.10, *p* = .003, partial η^2^ = .086, 95% CI = [−1.70, −0.35]. In addition, Black perceivers evaluated ambiguous acts of cultural appropriation (*M* = 3.99, *SD* = 1.93) as less harmful than overt acts (*M* = 5.84, *SD* = 1.51); *F* (1,97) = 47.26, *p* < .001, partial η^2^ = .33, 95% CI = [−0.23, −1.31]. This effect was also significant among White perceivers, who evaluated ambiguous acts of cultural appropriation (*M* = 2.97, *SD* = 1.43) as less harmful than overt acts (*M* = 5.56, *SD* = 1.51), *F* (1,97) =105.66, *p* < .001, partial η^2^ = .52; 95% CI = [−3.20, −2.16]; although the effect of ambiguity was stronger among White participants.

## Study 2

Study 1 supported initial predictions of differential perceptions of harm enacted toward the source community based on the racialization of the perceiver and the overtness of cultural appropriation, and provides evidence for the *aversive racism hypothesis of cultural appropriation (H1*): White perceivers saw less harm in acts of cultural appropriation than Black perceivers, but this was only the case when reading about an ambiguous act of cultural appropriation (e.g., literature). When reading about an overt act of cultural appropriation (e.g., culture parties), Black and White participants saw similar levels of harm. Study 2 sought to replicate this effect in a new sample, as well as investigate *the intent-as-justification hypothesis (H2)*: whether attributions of positive intent to actors engaged in ambiguous (vs. overt) acts of cultural appropriation explained differential evaluations of resulting intergroup harm among White and Black perceivers.

### Method

#### Participants

The sample size for this study was based on the same a priori power analysis as in Study 1. A total of 118 adults living in the United States were recruited via Prolific.com. A pre-screening survey was used to recruit Black and White American participants. Fourteen participants were recruited, but did not complete the study and thus were excluded. Eight participants were removed for reporting suspicion with materials (e.g., that they were “fake articles”). Six respondents were removed for not following instructions, and two participants were removed for reporting difficulty with understanding materials, resulting in a final analytic sample of 88 participants. Based on a sensitivity power analysis in G*Power, this sample size provided 0.80 power to detect an interaction effect of Participant Race × Appropriation Ambiguity of 0.18 or greater.

The 88 participants whose responses were included in analyses ranged from 20 to 76 years old (*M* = 40.46 years, *SD* = 14.34 years). Participants self-identified their race as White American (48.9%) or Black American (51.15%) and their gender as female (47.1%), male (52.9%). The hypotheses and materials for this study were preregistered at the Open Science Framework (https://osf.io/azqv8/?view_only=9dd40f0cae44480a8cc942de3eb007f5).

#### Design and Procedures

This study adopted the same 2 (Participant Race: White vs. Black—between) × 2 (Appropriation Ambiguity: Ambiguous vs. Overt—within) mixed-subjects design as in Study 1. The procedure for Study 2 was identical to Study 1, where participants learned that the purpose of the study was to “understand what people think about different social situations,” and read about both an *ambiguous* case of cultural appropriation and an *overt* case in a counterbalanced order. Scenarios in the “Ambiguous Appropriation Condition” and the “Overt Appropriation Condition” were the same as in Study 1 (Mosley & Biernat, 2021).

After reading each scenario, participants then indicated their *attributions of positive intent to the target* using three items (items adapted from Mosley & Biernat, 2021), including (a) “[Target] has good intentions,” (b) “[Target] has positive objectives,” and (c) “[Target] is intentionally trying to create good” (1—*Strongly Disagree*; 4—*Neither Agree nor Disagree*; 7—*Strongly Disagree*). For analyses below, three items were averaged for each condition (Ambiguous: α = .96; Overt: α = .96).

Participants then indicated their *evaluations of harm* enacted toward the source community using the same three items as Study 1 (Mosley & Biernat, 2021; Ambiguous: α = .96; Overt: α = .78).

Afterward, participants completed the same single-item *manipulation check* of perceived overtness of cultural appropriation as in Study 1 (Mosley, Heiphetz, et al., 2023). Participants then completed demographics and were debriefed.

### Results

#### Manipulation Check

First, to assess the effectiveness of the manipulation check, perceptions of cultural appropriation was analyzed using a 2 (Participant Race: White vs. Black—between) × 2 (Appropriation Ambiguity: Ambiguous vs. Overt—within) mixed-design ANOVA. Each effect was as follows: (a) Participant Race, *F* (1,85) = 5.02, *p* = .028, *partial* η^2^ = .056, indicating that Black participants (*M* = 4.60, *SD* = 1.56) perceived greater cultural appropriation, overall, compared with White participants (*M* = 3.98, *SD* = 1.74); (b) Appropriation Ambiguity, *F*(1,85) = 117.205, *p* < .001, *partial* η^2^ = .58; indicating that all participants evaluated the overt cultural appropriation scenario (i.e., Culture Party; *M* = 5.49, *SD* = 1.68) as more of an obvious act of cultural appropriation, on average, than the ambiguous scenario of cultural appropriation (i.e., Literature; *M* = 3.10, *SD* = 1.67); (c) Participant Race × Appropriation Ambiguity, *F* (1,85) = 0.027, *p* = .87, *partial* η^2^ < .001.

#### Perceived Harm

**Figure 2.**
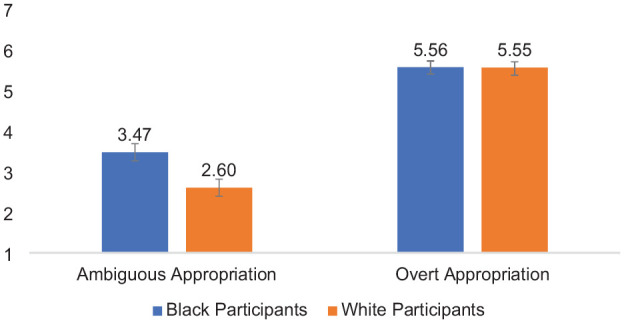
Participant Race × Appropriation Ambiguity Interaction on Evaluations of Harm Toward the Source Community, Study 2. *Note*. Error bars represent standard error bars.

Second, of most relevance to the main question of interest (whether Black and White participants evaluate harm as a result of ambiguous vs. overt forms of cultural appropriation), perceived harm was analyzed using a 2 (Participant Race: White vs. Black—between) × 2 (Appropriation Ambiguity: Ambiguous vs. Overt—within) mixed-design ANOVA. Each effect was as follows: (a) Participant Race, *F* (1,85) = 3.77, *p* = .056, *partial* η^2^ = .042, indicating that Black (*M* = 4.52, *SD* = 1.46) and White participants (*M* = 4.08, *SD* = 1.38) perceived similar levels of harm, on average; (b) Appropriation Ambiguity, *F* (1,85) = 28.79, *p* < .001, *partial* η^2^ = .94; indicating that all participants evaluated actors who engaged in overt cultural appropriation (*M* = 5.56, *SD* = 1.36) as more harmful, on average, than actors who engaged in ambiguous cultural appropriation (*M* = 3.05, *SD* = 1.54). Confirming the *aversive racism theory of cultural appropriation* (H1), the two-way interaction of Participant Race × Appropriation Ambiguity was significant, *F* (1,85) = 4.40, *p* = .04, *partial* η^2^ = .05 (see Figure 2). Means and standard deviations are shown in Table 2.

**Table 2.** Dependent Measures by Participant Race and Appropriation Ambiguity, Study 2.

	Participant race × Appropriation ambiguity			
	Ambiguous appropriation		Overt appropriation	
Measure	White participants	Black participants	White participants	Black participants
	*M (SD)*	*M (SD)*	*M (SD)*	*M (SD)*
Manipulation check	2.76 (1.53)	3.42 (1.75)	5.19 (1.94)	5.78 (1.36)
Positive actor intent	5.44 (1.31)	4.85 (1.46)	1.70 (1.10)	1.94 (1.41)
Evaluations of harm	2.60 (1.29)	3.47 (1.65)	5.55 (1.47)	5.56 (1.26)

Simple effects tests based on the interaction indicated when reading about ambiguous cultural appropriation, White perceivers (*M* = 2.60, *SD* = 1.28) evaluated the target as less harmful than Black perceivers (*M* = 3.47, *SD* = 1.65), *F* (1,85) = 7.49, *p* = .008, *partial* η^2^ = .08, 95% CI = [−1.50, −0.24]. When reading about overt appropriation, Black perceivers (*M* = 5.56, *SD* = 1.26) and White perceivers (*M* = 5.55, *SD* = 1.47) similarly evaluated the target on perceived harm, *F* (1,85) = 0.003, *p* = .96, *partial* η^2^ < .001, 95% CI = [−0.60, 0.57]. In addition, Black perceivers evaluated ambiguous acts of cultural appropriation (*M* = 3.47, *SD* = 1.64) as less harmful than overt acts (*M* = 5.56, *SD* = 1.36), *F* (1,85) = 54.33, *p* < .001, partial η^2^ = .39; 95% CI = [−2.65, −1.53]. White perceivers also evaluated ambiguous acts of cultural appropriation (*M* = 2.60, *SD* = 1.29) as less harmful than overt acts (*M* = 5.55, *SD* = 1.47), *F* (1,85) = 100.76, *p* < .001, partial η^2^ = .54, 95% CI = [−3.53, −2.36], although this effect was stronger among White perceivers than Black perceivers.

#### Perceived Target Intent

**Figure 3.**
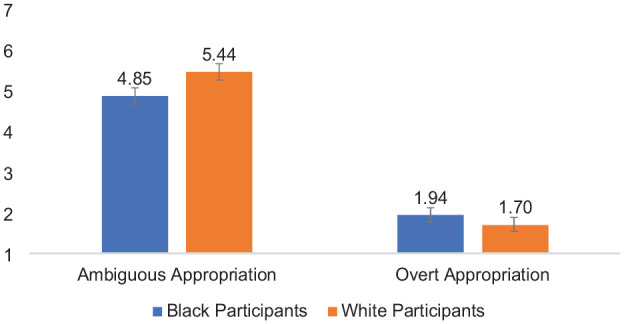
Participant Race × Appropriation Ambiguity Interaction on Attributions of Positive Intent to the Actor, Study 2. *Note*. Error bars represent standard error bars.

Third, I analyzed perceptions of target intent using a 2 (Participant Race: White vs Black—between) × 2 (Appropriation Ambiguity: Ambiguous vs. Overt—within) mixed-design ANOVA. Each effect was as follows: (a) Participant Race, *F* (1,85) = 0.62, *p* = .43, *partial* η^2^ = .007, indicating that Black perceivers (*M* = 3.40, *SD* = 1.43) and White perceivers (*M* = 3.57, *SD* = 1.21) delivered equal perceptions of positive intent, on average, when responding to acts of cultural appropriation; (b) Appropriation Ambiguity, *F* (1,85) = 322.00, *p* < .001, *partial* η^2^ = .79; indicating that participants evaluated actors who engaged in ambiguous cultural appropriation (*M* = 5.13, *SD* = 1.41) as more positively intentioned, on average, than actors who engaged in overt cultural appropriation (*M* = 1.82, *SD* = 1.27). Confirming H2 (*the intent-as-justification hypothesis*), the two-way interaction of Participant Race × Appropriation Ambiguity was significant, *F* (1,85) = 4.98, *p* = .03, partial η^2^ = .06 (see Figure 3). Means and standard deviations are shown in Table 2.

Simple effects tests based on the interaction indicated that when reading about ambiguous cultural appropriation, there was a trend that suggested White perceivers (*M* = 5.44, *SD* = 1.31) evaluated the target as more positively intentioned than Black participants (*M* = 4.85, *SD* = 1.46), *F* (1,85) = 3.83, *p* = .05, *partial* η^2^ = .04; 95% CI = [−0.009, 1.18]. When reading about overt appropriation, Black perceivers (*M* = 1.94, *SD* = 1.41) and White perceivers (*M* = 1.70, *SD* = 1.10) similarly evaluated the target on perceived positive intent, *F* (1,85) = 0.80, *p* = .38, partial η^2^ = .009; 95% CI = [−0.78, 1.29]. Furthermore, Black perceivers viewed the target engaged in overt cultural appropriation (*M* = 1.94, *SD* = 1.41) as less positively intentioned compared with the target engaged in ambiguous cultural appropriation (*M* = 4.85, *SD* = 1.46), *F* (1,85) = 127.85, *p* < .001, partial η^2^ = .06; 95% CI = [2.40, 3.42]. White perceivers also evaluated the target engaged in overt cultural appropriation (*M* = 1.70, *SD* = 1.10) as less positively intentioned than the target engaged in ambiguous cultural appropriation (*M* = 5.44, *SD* = 1.31), *F* (1,85) = 196.75, *p* < .001, partial η^2^ = .70, 95% CI = [3.21, 4.27]; however, the ambiguity effect was stronger among White participants than Black participants.

Next, I tested two separate mediation models to examine whether the Participant Race effect on perceptions of harm was mediated by perceived actor positive intent in the (a) ambiguous appropriation and (b) overt appropriation conditions.

For each model, I conducted a mediation analysis using the PROCESS macro for SPSS (Hayes, 2013, Model 4) with 5,000 bias-corrected bootstrap resamples. I regressed perceptions of harm on participant race (Coded 0 = White Participants, 1 = Black Participants), positive intent as the mediator.

**Figure 4.**
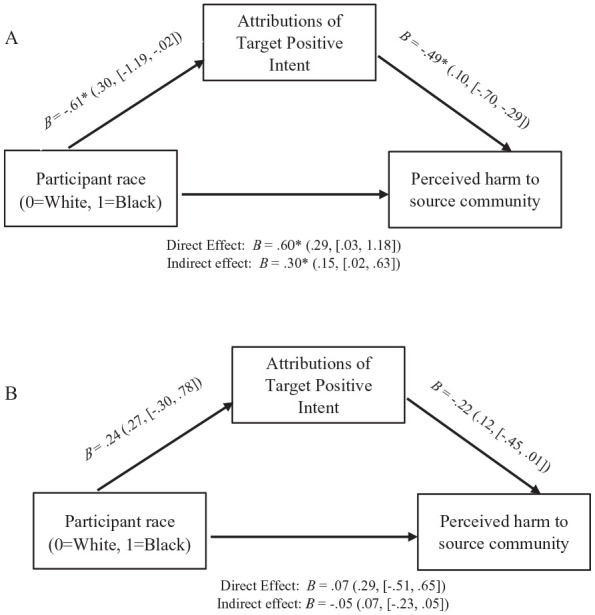
Simple Mediation Models: Attributions of Actor Positive Intent Mediates the Effect of Participant Race on Perceived Harm to the Source Community in the Ambiguous Cultural Appropriation Condition, Study 2 (SEs and [95% CIs] in Parentheses): (A) Simple Mediation Model, Ambiguous Cultural Appropriation Condition and (B) Simple Mediation Model, Overt Cultural Appropriation Condition.

Figure 4 depicts simple mediation of the participant race effect on perceived harm via positive actor intent in the ambiguous appropriation condition (Panel A) and the overt appropriation condition (Panel B).

As predicted, the conditional indirect effect of participant race on perceived harm, via positive actor intent, was significant in the ambiguous appropriation condition, indirect effect = 0.30, *SE* = 0.15, 95% CI = [0.02, 0.63], but was not significant in the overt appropriation condition, indirect effect *=* −0.05, *SE* = 0.07, 95% CI = [−0.23, 0.05].

## Study 3

Study 2 provided additional evidence for the *aversive racism theory of cultural appropriation (H1)*, such that White participants evaluated less harm than Black participants in the acts of ambiguous acts of cultural appropriation, but not in overt acts. Study 2 also provided evidence for the *intent-as-justification hypothesis* (H2), such that White participants would evaluate less harm in ambiguous acts of cultural appropriation because they attributed more positive intent to the target. However, the mixed design for Study 2 only allows for the analysis of conditional indirect effects via two separate simple mediation models (Model 4) using PROCESS (see Hayes, 2013; Montoya & Hayes, 2016). Thus, to investigate the analysis of conditional indirect effects with a single moderated mediation analysis (Model 8), Study 3 uses a fully between subject’s design. Participants read about White targets who (a) engaged in an ambiguous act of cultural appropriation (i.e., a White artist painting Black characters) *or* (b) engaged in an overt act of cultural appropriation (i.e., a White fraternity having a “Ghetto Party.”).

### Method

#### Participants

The sample size for this study was based on the effect sizes in Study 1 for two-way interaction of Participant Race × Appropriation Ambiguity (partial η^2^ = .05) and Study 2 (partial η^2^ = .05) using G*Power software (Faul et al., 2007). A power analysis that estimated a small effect size (*f* = 0.15), and included other standard parameters (α = .05, and 80% power) for a 2 × 2 between subjects’ analysis, estimated a desired sample size of 351 participants. I oversampled by 10% (36) to account for participant exclusions. Therefore, I aimed to collect a sample size of 388 participants (to be divisible by 4).

A total of 406 adults living in the United States were recruited via Prolific.com. A pre-screening survey was used to recruit Black and White American participants. Eighteen participants were recruited, but did not complete the study (not assigned to condition), thus were excluded. Four participants were removed for reporting a different ethnicity than the pre-screening. Eight participants requested to have their data removed at the end of the study. Ten respondents were removed for not following instructions, and 16 participants were removed for reporting difficulty with understanding materials, resulting in a final analytic sample of 350 participants. Based on a sensitivity power analysis in G*Power, this sample size provided 0.80 power to detect an interaction effect of Participant Race × Appropriation Ambiguity of 0.15 or greater.

The 350 participants whose responses were included in analyses ranged from 19 to 79 years old (*M* = 39.6 years, *SD* = 12.89 years). Participants self-identified their race as White American (49.4%) or Black American (50.6%) and their gender as female (44.9%), male (52.9%), and 2.3% reported “other gender.” The hypotheses and materials for this study was preregistered at the Open Science Framework (https://osf.io/xhtak/?view_only=852c6dc4345f4836bb372945c39d6074).

#### Design and Procedures

This study adopted a 2 (Participant Race: White vs. Black) × 2 (Appropriation Ambiguity: Ambiguous vs. Overt) between-subjects design. The procedure for Study 3 was similar to Studies 1 and 2, where participants learned that the purpose of the study was to “understand what people think about different social situations,” and read about both an *ambiguous* case of cultural appropriation and an *overt* case in a counterbalanced order.

Half of the participants were randomly assigned to read about an *ambiguous* case of cultural appropriation, and the other half of the participants were randomly assigned an *overt* case of cultural appropriation. The two conditions differed with respect to how culturally appropriative they were perceived to be in previous research, but were similar in terms of the ontological nature and the *type of appropriation* that occurred in that they both used subject appropriation (Young, 2010). Moreover, the vignettes were similar with respect to word count, and were taken directly from prior research that manipulated acts of cultural appropriation in a variety of scenarios (Mosley & Biernat, 2021; Mosley, Biernat, & Adams, 2023), and were analogous to other research that investigated acts of cultural appropriation using a more diverse set of scenarios (Mosley, Heiphetz, et al., 2023).

The “Ambiguous Appropriation Condition” described a new scenario about a White artist creating artwork based on the Black characters. Specifically, the ambiguous appropriation condition consistently scored the *low* in eight scenarios on perceptions of cultural appropriation in prior work (*M*_perceived appropriation_ ranged from 3.03 to 3.10 on a 7-point scale; Mosley & Biernat, 2021; Studies 1–3). Similarly, recent research demonstrates that perceivers tend to rate an analogous scenario as *particularly low* on perceptions of cultural appropriation, “*A White American artist is famous for painting African American subjects and stories*” relative to 56 other scenarios of outgroup cultural use (*M*_perceived appropriation_ ranged from 2.94 to 3.85 on a 7-point scale; Mosley, Heiphetz, et al., 2023; Studies 1–2).

The “Overt Appropriation Condition” described the same scenario in Studies 1 and 2 about a White fraternity throwing a “ghetto-themed” Compton Cookout party where party goers were encouraged to perform the subjective experiences and culture of Black people.

After reading each scenario, participants then indicated their *attributions of positive intent to the target* using the same three items as in Study 2 (items adapted from Mosley & Biernat, 2021). For analyses below, three items were averaged for each condition (overt α = .97; ambiguous α = .94).

Participants then indicated their *evaluations of harm* enacted toward the source community using the same three items as Studies 1 and 2 (Mosley & Biernat, 2021). For analyses below, three items were averaged for each condition (overt α = .88; ambiguous α = .93).

Afterward, participants completed the same single-item *manipulation check* of perceived overtness of cultural appropriation as in Studies 1 and 2 (Mosley, Heiphetz, White, & Biernat, 2023). Finally, participants then completed demographics and were debriefed. The supplementary materials include a complete list of the materials.^1^

### Results

#### Manipulation Check

In order to assess the effectiveness of the manipulation check, perceptions of cultural appropriation were analyzed using a 2 (Participant Race: White vs. Black) × 2 (Appropriation Ambiguity: Ambiguous vs. Overt) between subject’s design ANOVA. Each effect was as follows: (a) Participant Race, *F* (1,346) = 41.29, *p* < .001, *partial* η^2^ = .11, indicating that Black participants (*M* = 5.29, *SD* = 1.60) judged the scenarios as more overtly appropriative, overall, compared with White participants (*M* = 4.06, *SD* = 2.10); (b) Appropriation Ambiguity, *F*(1,346) = 20.62, *p* < .001, *partial* η^2^ = .056; indicating that all participants evaluated the overt cultural appropriation scenario (i.e., Culture Party; *M* = 5.11, *SD* = 1.91) as more of an obvious act of cultural appropriation, on average, than the ambiguous scenario of cultural appropriation (i.e., Art; *M* = 4.21, *SD* = 1.91); (c) Participant Race × Appropriation Ambiguity, *F* (1,346) = 5.68, *p* = .018, *partial* η^2^ = .016.

Simple effects tests based on the interaction indicated that when reading about overt cultural appropriation, Black perceivers (*M* = 5.49, *SD* = 1.62) evaluated the scenario as more blatantly appropriative than White perceivers (*M* = 4.71, *SD* = 2.12), *F* (1,346) = 8.56, *p* = .004, partial η^2^ = .024, 95% CI = [−1.31, −0.26]. When reading about ambiguous appropriation, Black perceivers (*M* = 5.07, *SD* = 1.56) evaluated the scenario as more blatantly appropriative than White perceivers (*M* = 3.37, *SD* = 1.86), *F* (1,346) = 37.12, *p* < .001, partial η^2^ = .10, 95% CI = [−2.25, −1.15]. In addition, White perceivers evaluated the ambiguous act of cultural appropriation as less blatant than the overt act, *M_ambiguous_* = 3.37, *SD_ambiguous_* = 1.86; *M_overt_* = 4.71, *SD_overt_* = 2.12; *F* (1,346) = 23.74, *p* < .001, *partial* η^2^ = .064, 95% CI = [−1.89, −0.80], but Black participants did not *M_ambiguous_* = 5.07, *SD_ambiguous_* = 1.56; *M_overt_* = 5.49, *SD_overt_* = 1.62; *F* (1,346) = 2.35, *p* = .126, *partial* η^2^ = .007, 95% CI = [−0.95, 0.12].

#### Perceived Harm

**Figure 5.**
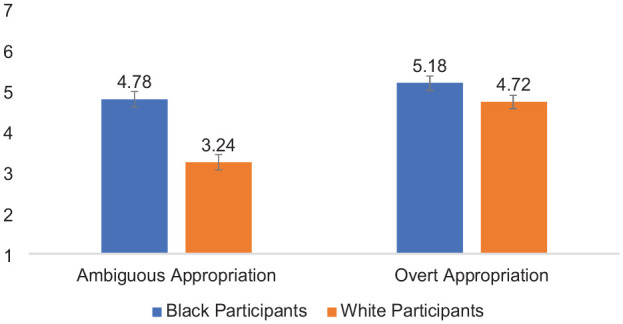
Participant Race × Appropriation Ambiguity Interaction on Evaluations of Harm Toward the Source Community, Study 3. *Note*. Error bars represent standard error bars.

Perceived harm was analyzed using a 2 (Participant Race: White vs. Black) × 2 (Appropriation Ambiguity: Ambiguous vs. Overt) between-subjects design ANOVA. Each effect was as follows: (a) Participant Race, *F* (1,346) = 28.91, *p* < .001, *partial* η^2^ = .077, indicating that Black participants (*M* = 5.00, *SD* = 1.68) perceived greater harm overall compared with White participants (*M* = 4.00, *SD* = 1.96); (b) Appropriation Ambiguity, *F* (1,346) = 25.34, *p* < .001, *partial* η^2^ = .068; indicating that all perceivers evaluated the overt cultural appropriation scenario (*M* = 4.96, *SD* = 1.81) as more harmful, on average, than the ambiguous cultural appropriation scenario (*M* = 4.00, *SD* = 1.84). Providing further evidence for the *aversive racism theory of cultural appropriation* (H1), the two-way interaction of Participant Race × Appropriation Ambiguity was significant, *F* (1,346) = 8.34, *p* = .004, *partial* η^2^ = .024 (see Figure 5). Means and standard deviations are shown in Table 3.

**Table 3.** Dependent Measures by Participant Race and Appropriation Ambiguity, Study 3.

	Participant race × Appropriation ambiguity			
	Ambiguous appropriation		Overt appropriation	
Measure	White participants	Black participants	White participants	Black participants
	*M (SD)*	*M (SD)*	*M (SD)*	*M (SD)*
Manipulation check	3.37 (1.86)	5.07 (1.56)	4.71 (2.12)	5.49 (1.62)
Positive actor intent	4.40 (1.37)	3.70 (1.60)	2.28 (1.57)	2.60 (1.93)
Evaluations of harm	3.24 (1.77)	4.78 (1.58)	4.72 (1.86)	5.18 (1.74)

Probing the interaction, simple effects tests revealed that when reading about ambiguous cultural appropriation, White perceivers (*M* = 3.24, *SD* = 1.77) evaluated the target as less harmful than Black perceivers (*M* = 4.78, *SD* = 1.59), *F* (1,346) = 32.67, *p* < .001, *partial* η^2^ = .086, 95% CI = [−2.08, −1.01]. When reading about overt appropriation, Black perceivers (*M* = 5.18, *SD* = 1.74) and White perceivers (*M* = 4.72, *SD* = 1.86) similarly evaluated harm, *F* (1,346) = 3.25, *p* = .073, partial η^2^ = .009, 95% CI = [−0.97, 0.04]. In addition, Black perceivers evaluated the ambiguous act of cultural appropriation (*M* = 4.78, *SD* = 1.58) as similarly harmful as the overt act (*M* = 5.18, *SD* = 1.74), *F* (1,346) = 2.33, *p* = .128, *partial* η^2^ = .007, 95% CI = [−0.92, 0.12]. However, White perceivers evaluated the ambiguous act of cultural appropriation (*M* = 3.24, *SD* = 1.77) as less harmful than the overt act (*M* = 4.72, *SD* = 1.86), *F* (1,346) = 31.07, *p* < .001, *partial* η^2^ = .082, 95% CI = [−2.00, −0.96].

#### Perceived Target Intent

**Figure 6.**
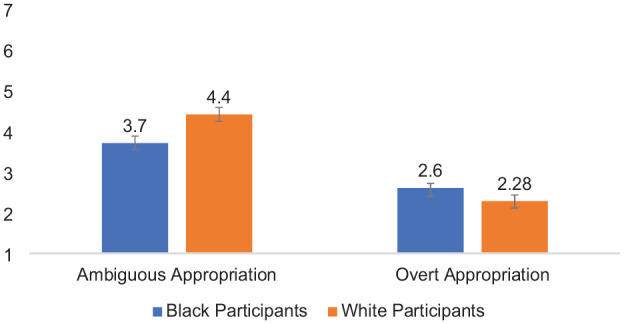
Participant Race × Appropriation Ambiguity Interaction on Attributions of Positive Intent to the Actor, Study 3. *Note*. Error bars represent standard error bars.

I then analyzed perceptions of target intent using a 2 (Participant Race: White vs. Black) × 2 (Appropriation Ambiguity: Ambiguous vs. Overt) between-subjects design ANOVA. Each effect was as follows: (a) Participant Race, *F* (1,346) = 1.13, *p* = .29, *partial* η^2^ = .003, indicating that Black perceivers (*M* = 3.12, *SD* = 1.86) and White perceivers (*M* = 3.31, *SD* = 1.81) delivered equal perceptions of positive intent, on average, when responding to acts of cultural appropriation; (b) Appropriation Ambiguity, *F* (1,346) = 84.15, *p* < .001, *partial* η^2^ = .20; indicating that all perceivers evaluated actors who engaged in ambiguous cultural appropriation (*M* = 4.05, *SD* = 1.52) as more positively intentioned, on average, than actors who engaged in overt cultural appropriation (*M* = 2.45, *SD* = 1.77). Providing additional evidence for *the intent-as-justification hypothesis (H2*), the two-way interaction of Participant Race × Appropriation Ambiguity was significant, *F* (1,346) = 8.40, *p* = .004, *partial* η^2^ = .024 (see Figure 6). Means and standard deviations are shown in Table 3.

Probing the interaction, simple effects indicated that when reading about ambiguous cultural appropriation, White perceivers (*M* = 4.40, *SD* = 1.37) evaluated the target as more positively intentioned than Black perceivers (*M* = 3.71, *SD* = 1.60), *F* (1,346) = 7.50, *p* = .006, *partial* η^2^ = .021, 95% CI = [0.20, 1.19]. When reading about overt appropriation, Black perceivers (*M* = 2.60, *SD* = 1.93) and White perceivers (*M* = 2.28, *SD* = 1.57) similarly evaluated the target on positive intent, *F* (1,346) = 1.77, *p* = .19, partial η^2^ = .005, 95% CI = [−0.80, 0.15]. In addition, Black perceivers evaluated the actor engaged in overt cultural appropriation (*M* = 2.60, *SD* = 1.93) as less positively intentioned than the target engaged in ambiguous cultural appropriation (*M* = 3.70, *SD* = 1.60), *F* (1,346) = 19.89, *p* < .001, partial η^2^ = .054, 95% CI = [0.62, 1.59]. White perceivers also evaluated the target engaged in ambiguous acts of cultural appropriation (*M* = 4.40, *SD* = 1.37) as less positively intentioned than the target engaged in the overt act (*M* = 2.28, *SD* = 1.57), *F* (1,346) = 72.14, *p* < .001, partial η^2^ = .17, 95% CI = [1.63, 2.61]. Once again, the ambiguity effect was stronger among White participants than Black participants.

**Figure 7.**
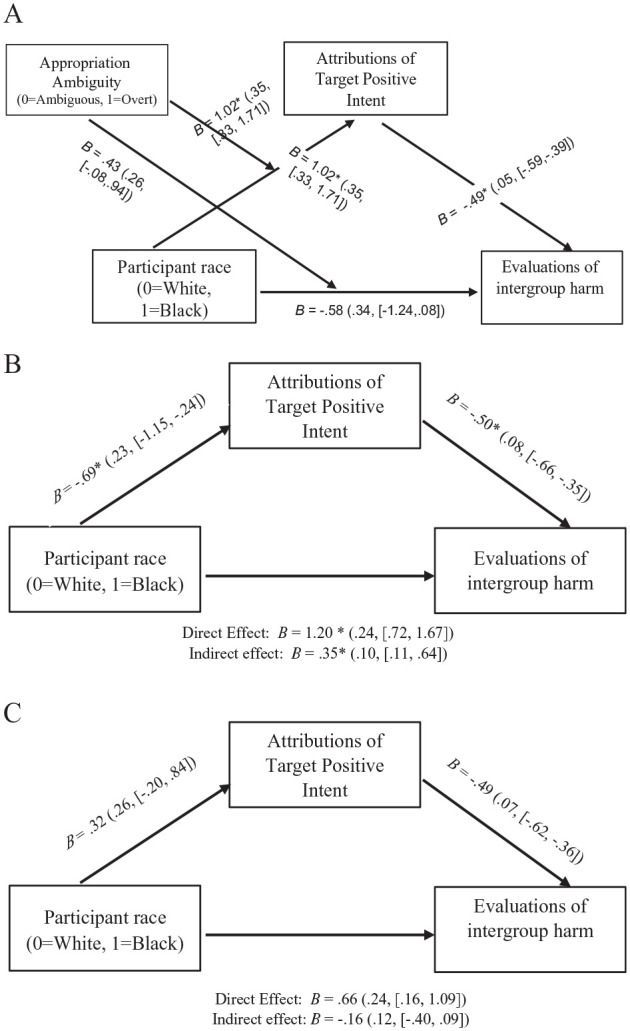
Moderated Mediation Model: Attributions of Positive Intent Mediates the Participant Race by Appropriation Ambiguity Interaction on Perceived Harm to the Source Community, Study 3 (SEs and [95% CIs] in Parentheses). (A) Overall moderated mediation model. (B) Simple mediation model, ambiguous cultural appropriation condition. (C) Simple mediation model, overt cultural appropriation condition.

I then tested the moderated mediation model depicted in Figure 7 (Panel A) to examine whether the Participant Race × Appropriation Ambiguity interaction on perceptions of harm was mediated by perceived intent. I conducted a moderated mediation analysis using the PROCESS macro for SAS (Hayes, 2013, Model 8) with 5,000 bias-corrected bootstrap resamples. I regressed perceptions of harm on participant race (Coded 0 = White Participants, 1 = Black Participants), appropriation ambiguity (Coded 0 = Ambiguous Appropriation, 1 = Overt Appropriation), and their interaction, with perceived intent as the mediator. Appropriation ambiguity was included as a moderator variable of the *a* path (i.e., participant race to perceived harm) and the *c* path (i.e., participant race to perceived harm).

The index of moderated mediation was significant, effect = −0.50, *SE* = 0.18, 95% CI = [−0.86, −0.16]. The conditional indirect effect of participant race on perceived harm, via perceived intent, was not significant in the overt appropriation condition, indirect effect = −0.16, *SE* = 0.13, 95% CI = [−0.41, 0.10], but was significant in the ambiguous appropriation condition, indirect effect *=* 0.34, *SE* = 0.12, 95% CI = [0.12, 0.60]. Figure 7 also depicts simple mediation of the participant race effect on perceived harm via perceived intent in the ambiguous appropriation condition (Panel B) and the overt appropriation condition (Panel C). In both the ambiguous appropriation condition and overt appropriation condition, White participants (compared with Black participants) attributed more positive intent to the target, which in turn predicted perceptions of harm.

In a comparable model with appropriation ambiguity as the predictor and participant race as the moderator, the conditional indirect effect of appropriation ambiguity on perceived harm, via perceived intent, was significant when perceivers were White, indirect effect = 1.05, *SE* = 0.16, 95% CI = [−0.74, 1.36], and when perceivers were Black, indirect effect = 0.54, *SE* = 0.18, 95% CI = [−0.56, −0.16], although the effect was stronger among White perceivers.

## Study 4

In a fully between-subjects design using a new scenario of ambiguous cultural appropriation (art), Study 3 replicated the finding that appropriation ambiguity and perceiver race interactively affected perceptions of intergroup harm. Studies 2 and 3 allowed for the measurement of naturally occurring variation in attributions of positive intent but did not allow for causal conclusions in an experimental design. Therefore, Study 4 manipulated attributions of target intent to examine evaluations of harm when there is (a) a focus on positive intent, (b) negative intent, and (c) no mention of intent in an ambiguous form of cultural appropriation. Overt appropriation was not assessed in this study. Study 4 used a new scenario of outgroup cultural use to represent an ambiguous form of cultural appropriation in the domain of object appropriation, which refers to a concrete physical cultural object (Young, 2010): a White chef who opens up a restaurant cooking soul food (a Black cultural product). Associated with harm is the protection and the engagement of collective action. If one does not believe that the source community has rights to collective ownership, they also less likely to protect them from cultural theft. Thus, Study 4 also investigated downstream consequences of collective action to fight discrimination against the source community (van Zomeren et al., 2004).

### Method

#### Participants

The sample size for this study was based on an a priori power analysis using G*Power software that conservatively assumed a small effect based on prior research on differences in cognition across perceiver status (e.g., dominant vs. minoritized) and perceptions of target intent (e.g., Swim et al., 2003). Conservatively assuming a small effect size (*f* = 0.15), α = .05, and 80% power for a 2 × 3 between subjects’ analysis, the power analysis estimated a desired sample size of 351 participants. I oversampled by 10% (36) to account for participant exclusions. Therefore, I aimed to collect a sample size of 388 participants (to be divisible by 4).

A total of 405 adults living in the United States were recruited via Prolific.com. A pre-screening survey was used to recruit Black and White American participants. Twenty-six participants were recruited, but did not complete the study and thus were excluded. Eleven respondents were removed for self-identifying as members of a different racial group during the demographics portion of the study. Nine participants were removed for duplicate IP addresses (first responses were maintained), and eight participants were removed for reporting difficulty understanding materials. Five were removed for reporting suspicion with materials (e.g., that materials were fake), and 13 participants were removed for failing to follow instructions, resulting in a final analytic sample of 333 participants. Based on a sensitivity power analysis in G*Power, this sample size provided 0.80 power to detect an interaction effect of Participant Race × Intent of 0.15 or greater.

The 333 participants whose responses were included in analyses ranged from 18 to 94 years old (*M* = 41.01 years, *SD* = 14.32 years). Participants self-identified their race as White American (51.7%) or Black American (48.3%) and their gender as female (50.5%), male (47.7.6%), <1% self-identified as nonbinary, and <1% self-identified as agender.

The hypotheses and materials for this study was preregistered at the Open Science Framework (OSF; https://osf.io/awfe7).

Upon acceptance, the following will be posted in the corresponding author’s OSF repository (a) materials, (b) analysis code, (c) datafile, and (d) codebook for interpreting the data file(s) describing all variables and how they are coded and link to them in the manuscript.

#### Design and Procedures

This study adopted a 2 (Participant Race: White vs. Black) × 3 (Target Intent: Positive Intent vs. Control vs. Negative Intent) between-subjects design. The procedure for Study 3 was similar to Studies 1 and 2, where participants learned that the purpose of the study was to “understand what people think about different social situations,” except in the study, participants *only* read about an *ambiguous* case of cultural appropriation. This scenario was used in previous research manipulating cultural exploitation in the domain of cuisine (*M*_perceived appropriation_ = 3.62 on a 7-point scale in Mosley & Biernat, 2021; Study 5) and was specifically chosen due to its level of ambiguity relative to previously used scenarios.

Participants were asked to read a review about a “new trendy restaurant, Nick’s Soul Food Renaissance” and a menu that featured photos of menu items that represented Black cuisine (e.g., fried chicken and waffles, fried chitlins, black-eyed peas), as well as a photo and information about the chef. Participants then read about the chef’s response to an interview, which induced the target intent manipulation that was based on previous research manipulating target intent (Cushman, 2008; Swim et al., 2003). The supplementary materials include a complete list of the materials.

Participants were randomly assigned to either (a) a *positive intent condition, (b) negative intent condition, or (c) a control condition.*

In the *positive intent condition (n = 113)*, participants read that the chef responded,

In my craft, I only have the most positive intentions and honorable motivations to honor Blacks and their culture, as society does not take them seriously enough. In the curation of my cuisine, I only meant to celebrate soul food. Throughout my career, I only have intended to create good for the Black community, with the ultimate goal of paying homage to Black culture.

In the *negative intent condition (n = 109)*, participants read that the chef responded,

In my craft, I tend to have aggressively competitive intentions and less than honorable motivations. Many Blacks take themselves and their culture too seriously. In the curation of my cuisine, I have intentionally tried to show how my food is superior to other soul food chefs. Throughout my career, I only intended to the take advantage of every opportunity, with the ultimate goal of dominating the soul food industry.

In the *control condition (n = 111)*, participants read that the chef responded,

In my craft, I only use fresh, local ingredients and the finest cooking practices. Dishes that contain every possible flavor, sweet and sour, bitter, and fresh. Throughout my career, I have served the most delicious and popular culinary creations. Ingredients get a refined style. As a chef, I add my personal touch with a predilection for fresh tones, acids, and spices. Recognizable flavors are brought in an unexpected way.

After reading each scenario, participants then indicated their *evaluations of harm* enacted toward the source community using the same three items as Studies 1 and 2 (Mosley & Biernat, 2021; α = .93).

After rating evaluations of harm, participants completed a manipulation check of perceived intent, using the same three items as Studies 1 to 3 (items adapted from Mosley & Biernat, 2021). For analyses below, higher scores indicate greater attributions of positive intent to the target. Participants also completed a measure of *collective action* using five items (adapted from Mosley & Branscombe, 2020; van Zomeren et al., 2004; α = .94), including (a) “I am motivated to confront future anti-Black discrimination,” (b) “I feel obligated to engage in collective action to fight anti-Black discrimination,” (c) “I would do something together with other people to call attention to anti-Blackness in society,” (d) “I would participate in a protest with other people to stop anti-Black discrimination,” and (e) “I would support changes in policies that advocate for Black people’s rights” (1—*Strongly Disagree*; 4—*Neither Agree nor Disagree*; 7—*Strongly Disagree*). For analyses below, higher scores indicate intentions for collective action to fight anti-Black discrimination.

Participants also completed two items to items as an individual difference measure of *perceived discrimination against Black people* (adapted from Mosley & Branscombe, 2020; Schmitt, & Branscombe, 2002; α = .97), which served as a covariate for collective action, including, “Black people as a group have been unjustly victimized by society,” and “Black people as a group have been unfairly victimized because of their race” (1—*Strongly Disagree*; 4—*Neither Agree nor Disagree*; 7—*Strongly Disagree*).

Finally, participants then completed demographics, including a measure of political orientation (1—*Strongly Liberal*; 9—*Strongly Conservative*) that also served as a covariate for collective action and were debriefed.

### Results

#### Manipulation Check

First, to assess the effectiveness of the manipulation check, scores on attributions of positive intent of cultural appropriation were analyzed using a 2 (Participant Race: White vs. Black) × 3 (Target Intent: Positive Intent vs. Negative Intent vs. Control) between-subjects ANOVA. Each effect was as follows: (a) Participant Race, *F* (1,327) = 11.28, *p* < .001, partial η^2^ = .03, indicating that White perceivers (*M* = 4.67, *SD* = 2.04) attributed more positive intent to the target, overall, compared with Black perceivers (*M* = 4.02, *SD* = 2.17); (b) Intent, *F*(1,327) = 100.65, *p* < .001, *partial* η^2^ = .38; indicating that all perceivers attributed greater positive intent to those in the positive intent condition (*M* = 5.61, *SD* = 1.41) compared with those in the negative intent condition (*M* = 2.55, *SD* = 2.00; *p* < .001) and compared with those in the control condition (*M* = 4.84, *SD* = 1.61; *p* < .001); perceivers attributed greater positive intent to those in the control condition (*M* = 4.84, *SD* = 1.61) than those in the negative intent condition (*M* = 2.55, *SD* = 2.00), *p* < .001; (c) Participant Race × Target Intent, *F* (1,327) = 0.91, *p* = .40, partial η^2^ = .006.

#### Perceived Harm

**Figure 8.**
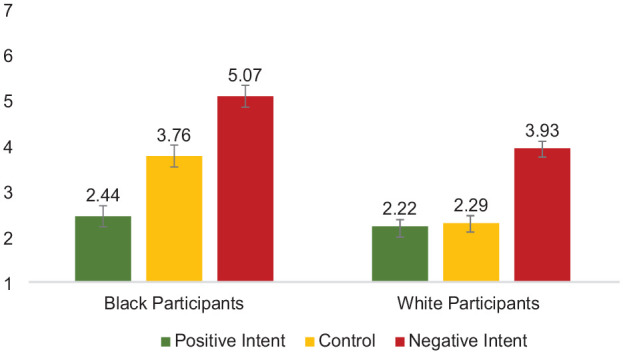
Participant Race × Target Intent Interaction on Evaluations of Harm, Study 4. *Note*. Error bars represent standard error bars.

Second, of most relevance to the primary question of interest (how Black and White participants evaluate harm as a result of different attributions of target intent), perceived harm was analyzed using a 2 (Participant Race: White vs. Black—between) × 3 (Target Intent: Positive Intent vs. Negative Intent vs. Control) between-subjects ANOVA. Each effect was as follows: (a) Participant Race, *F* (1,327) = 23.19, *p* < .001, partial η^2^ = .07, indicating that White perceivers (*M* = 2.79, *SD* = 1.93) evaluated less harm, overall, compared with Black perceivers (*M* = 3.76, *SD* = 2.09); (b) Target Intent, *F*(1,327) = 42.37, *p* < .001, partial η^2^ = .21; indicating that all perceivers evaluated greater harm in the negative intent condition (*M* = 4.49, *SD* = 2.08) compared with those in the positive intent condition (*M* = 2.32, *SD* = 1.61; *p* < .001) and compared with those in the control condition (*M* = 2.99, *SD* = 1.87; *p* < .001); perceivers attributed greater harm to those in the control condition than those in the positive intent condition, *p =* .04; (c) confirming the *intent-as justification hypothesis* (H2), the predicted two-way interaction of Participant Race × Intent was significant, *F* (1,327) = 3.71, *p* = .026, *partial* η^2^ = .022 (see Figure 8). Means and standard deviations are shown in Table 4.

**Table 4.** Dependent Measures by Participant Race and Target Positive Intent, Study 4.

	Participant race × Appropriation ambiguity					
	Positive intent		Control		Negative intent	
Measure	White participants	Black participants	White participants	Black participants	White participants	Black participants
	*M (SD)*	*M (SD)*	*M (SD)*	*M (SD)*	*M (SD)*	*M (SD)*
Manipulation check	5.85 (1.25)	5.35 (1.54)	5.30 (1.37)	4.35 (1.71)	2.74 (1.93)	2.36 (2.06)
Evaluations of harm	2.22 (1.60)	2.44 (1.63)	2.29 (1.49)	3.76 (1.95)	3.93 (2.17)	5.07 (1.83)
Collective action	4.58 (1.94)	5.53 (1.49)	4.81 (1.77)	5.36 (1.29)	4.45 (1.71)	5.82 (1.16)

Simple effects tests based on the interaction indicated that among participants who read about positive intent, White perceivers (*M* = 2.22, *SD* = 1.60) evaluated harm similarly as Black perceivers (*M* = 2.44, *SD* = 1.63), *F* (1,327) = 0.42, *p* = .52, *partial* η^2^ = .001, 95% CI = [−0.88, 0.44].

Among participants in the control condition, White perceivers (*M* = 2.29, *SD* = 1.49) evaluated less harm than Black perceivers (*M* = 3.76, *SD* = 1.95), *F* (1,327) = 18.82, *p* < .001, *partial* η^2^ = .054, 95% CI = [−2.14, −0.81].

Among participants who read about negative intent, White perceivers (*M* = 3.93, *SD* = 2.17) evaluated less harm than Black perceivers (*M* = 5.07, *SD* = 1.82), *F* (1,327) = 11.10, *p* < .001, *partial* η^2^ = .03, 95% CI = [1.81, −0.47].

The intent effect was significant among Black perceivers, *F* (1,327) = 29.22, *p* < .001, partial η^2^ = .15: those who read about negative intent (*M* = 5.07, *SD* = 1.83) evaluated more harm than those who read about positive intent (*M* = 2.44, *SD* = 1.63; *p* < .001, 95% CI = [1.95, 3.31]) and evaluated more harm than those in the control condition (*M* = 3.76, *SD* = 1.95; *p* < .001, 95% CI = [0.63, 1.99]); Black participants in the control condition (*M* = 3.76, *SD* = 1.95) evaluated more harm than those in the positive intent condition (*M* = 2.44, *SD* = 1.63) (*p* < .001, 95% CI = [0.64, 2.00]).

The intent effect was also significant among White perceivers, *F* (1,327) = 16.42, *p*<.001, *partial* η^2^ = .09: those who read about negative intent (*M* = 3.93, *SD* = 2.17) evaluated more harm than those who read about positive intent (*M* = 2.22, *SD* = 1.60; *p* < .001; 95% CI = [1.05, 2.37]) and more harm than those in the control condition (*M* = 2.29, *SD* = 1.49; *p* < .001; 95% CI = [0.98, 2.30]); White perceivers in the control condition (*M* = 2.29, *SD* = 1.49) evaluated similar levels of harm as those in the positive intent condition (*M* = 2.22, *SD* = 1.60; *p* = .84; 95% CI = [−0.58, 0.72]).

#### Collective Action

Third, to examine downstream behavioral consequences of harm perceptions (whether Black and White perceivers engage in collective action as a result of different attributions of target intent), collective action was analyzed using a 2 (Participant Race: White vs. Black—between) × 3 (Target Intent: Positive Intent vs. Negative Intent vs. Control) between-subjects ANOVA with perceived discrimination (*M* = 5.75, *SD* = 1.59) and political orientation (*M* = 3.74, *SD* = 2.14) entered as covariates, as previous work on cultural appropriation shows that the more conservative the respondents are, the weaker the relationship between perceptions of source community harm and perceptions of cultural appropriation (Mosley, Heiphetz, et al., 2023).

**Figure 9.**
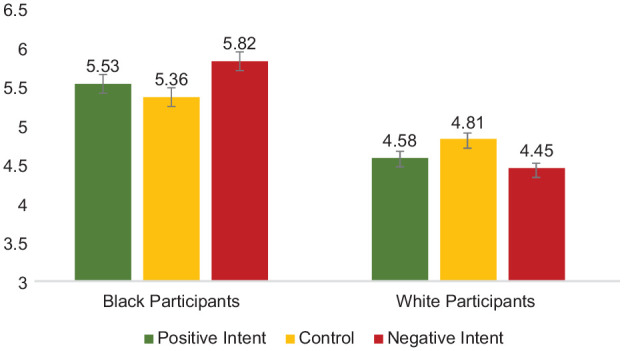
Participant Race × Target Intent Interaction on Collective Action, Study 3. *Note*. Error bars represent standard error bars.

Each effect was as follows: (a) Participant Race, *F* (1,325) = 16.07, *p* < .001, *partial* η^2^ = .05, indicating that White perceivers (*M* = 4.12, *SD* = 1.80) were less willing to engage in collective action, overall, compared with Black perceivers (*M* = 5.57, *SD* = 1.32); (b) Target Intent, *F*(1,325) = 0.26, *p* = .77, *partial* η^2^ = .002; indicating that, overall, there were no differences between those in the negative intent condition (*M* = 5.13, *SD* = 1.61), those in the positive intent condition (*M* = 5.03, *SD* = 1.79; *p* < .001), and those in the control condition (*M* = 5.07, *SD* = 1.57); (c) providing additional support for the *intent-as justification hypothesis* (H2), the predicted two-way interaction of Participant Race × Target Intent was significant, *F* (1,325) = 3.14, *p* = .045, *partial* η^2^ = .019 (see Figure 9). Means and standard deviations are shown in Table 4.

Simple effects tests based on the interaction indicated that among participants who read about positive intent, White perceivers (*M* = 4.58, *SD* = 1.94) were less willing to engage in collective action compared with Black perceivers (*M* = 5.53, *SD* = 1.49), *F* (1,325) = 7.90, *p* = .005, *partial* η^2^ = .024, 95% CI = [−1.10, −.20].

Among those in the control condition, White perceivers (*M* = 4.81, *SD* = 1.77) were similarly willing to engage in collective action as Black perceivers (*M* = 5.36, *SD* = 1.29), *F* (1,325) = .20, *p* = .658, *partial* η^2^ = .001, 95% CI = [−.57, .36].

Among those who read about negative intent, White perceivers (*M* = 4.45, *SD* = 1.71) were less willing to engage in collective action compared with Black perceivers (*M* = 5.82, *SD* = 1.16), *F* (1,325) = 14.79, *p* < .001, *partial* η^2^ = .04, 95% CI = [−1.38, −0.45].

Among Black perceivers, there was no effect of intent on collective action; *F* (1,325) = 2.27, *p* = .11, *partial* η^2^ = .01; negative intent condition (*M* = 5.82, *SD* = 1.16), positive intent condition (*M* = 5.53, *SD* = 1.49), and control condition (*M* = 5.36, *SD* = 1.29).

Among White perceivers, there was no effect of intent on collective action, *F* (1,325) = 1.08, *p* = .34, *partial* η^2^ = .007; negative intent condition (*M* = 4.45, *SD* = 1.71); positive intent (*M* = 4.58, *SD* = 1.94); and control condition (*M* = 4.81, *SD* = 1.77).

#### Perceived Harm on Collective Action

Finally, to investigate whether Black and White participants differentially engage in collective action as a result of perceived harm, collective action was regressed on Participant Race (0 = *White participants*, 1 = *Black participants*), Perceived Harm (*M* = 3.26; *SD* = 2.06), centered, and their interaction (see Table 4). The predicted interaction emerged such that perceived harm predicted greater collective action among Black participants, as well as White participants, and the tendency for Black participants to engage in greater collective action compared with White participants was significant among those who perceived low harm, those who perceived moderate harm, but not among those who perceived high perceived harm.

**Figure 10.**
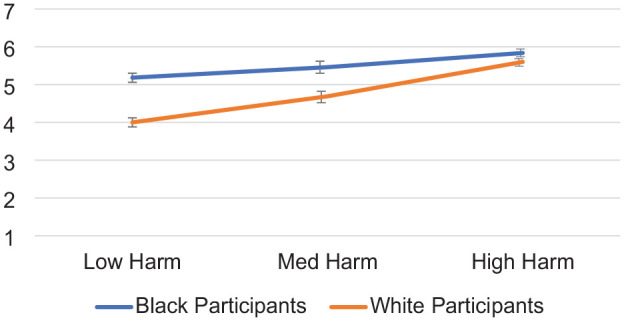
Summary of Participant Race × Perceived Harm effects on Collective Action, Study 4. *Note*. Error bars represent standard error bars.

Using the average of perceived harm, the effect of Participant Race was significant: At mean levels of perceived harm, Black perceivers were more likely to engage in collective action than White perceivers, *b* = 0.72, 95% CI = [0.75, 2.00], *SE* = 0.17, *t*(329) = 4.25 *p* < .001. The predicted interaction was also significant, *b* = −2.42, 95% CI = [−0.75, −0.08], *SE* = 0.17, *t*(329) = −2.42, *p* = .0061, and is displayed in Figure 10. Perceived harm predicted greater collective action among Black perceivers, *b* = 0.30, 95% CI = [0.06, 0.53], *SE* = 0.12, *t* (329) = 2.51, *p* = .012, and among White perceivers, *b* = 0.71, 95% CI = [0.46, 0.95], *SE* = 0.12, *t* (329) = 5.73, *p* < .001, although the effect is stronger among White perceivers. In addition, Black perceivers were more likely to engage in collective action than White perceivers among those who perceived low levels of harm (at the 16th percentile), *b* = 1.17, 95% CI = [0.68, 1.67], *SE* = 0.25, *t* (329) = 4.66, *p* < .0001, but not among those who perceived high levels of harm (at the 84th percentile), *b* = 0.024, 95% CI = [−0.28, 0.76], *SE* = 0.26, *t* (329) = 0.92, *p* = .36. The Johnson-Neyman test indicated that the Participant Race effect (the tendency to for Black perceivers to engage in more collective action than White perceivers) was significant at levels of perceived harm up to the 72.34 percentile (0.74 on the mean centered perceived harm index), suggesting that White perceivers only engage in similar levels collective action as Black perceivers when harm is blatantly obvious (in line with the aversive racism hypothesis, H1; see Table 5).

**Table 5.** Summary of Participant Race × Perceived Harm effects on Collective Action, Study 4.

Participant race (effect of participant race at mean level of harm)	Participant race × Harm interaction^a^	Simple effects (*b*s and significance)
*b* = 0.72***, *SE* = 0.17, 95% CI [0.39, 1.06]	*b* = −0.41*, *SE* = 0.17, 95% CI [−0.75, −0.08]	Harm among White: 0.71**** *Harm among Black: 0*.30 * *PR in Low*: 1.17**** *PR in High: 0.25*, *ns*

*Note.* Harm in White is the effect of Perceived Harm when the participants were White, Harm in Black is the effect of Perceived Harm when the participants were Black. PR in Low is the effect of Participant Race when Perceived Harm was low at the 16th percentile), PR in high is the effect of Participant Race when Perceived Harm was high (at the 84th percentile).

a The main effect of perceived harm is not directly interpretable in these analyses, as it represents the effect of perceived harm when Participant Race = 0 (White).

* *p* < .05. **p < .01. ***p < .001. ****p < .0001.

## Internal Meta-Analysis

Across studies and dependent measures, I consistently found evidence for Participant Race × Appropriation Ambiguity interactions. However, meta-analysis allows researchers to examine cumulative information across studies to gain a more accurate effect size than examining effect sizes within each study (Cumming, 2014). Thus, to obtain a precise estimate effect of perceived harm and perceived intent, to synthesize results, I conducted an internal meta-analysis across the four studies, testing particularly for the overall effect size of the two key predictions: The *aversive racism hypothesis* (when reading about ambiguous acts of cultural appropriation, are Black perceivers more likely to evaluate intergroup harm than White perceivers; H1), and the *intent as justification hypothesis* (when reading about ambiguous acts of cultural appropriation, are White perceivers more likely to attribute positive intent to targets than Black perceivers; H2). For the analysis of perceived harm, each study contributed one estimate for each effect described above from Studies 1 to 4 (using the control condition in Study 4). I also examined the effects of appropriation ambiguity (ambiguous appropriation vs. overt cultural appropriation) in Black and White perceivers, for three effects in each of four studies representing the simple effects in the 2 (participant race) × 2 (appropriation ambiguity) design. For the analysis of attributions of intent, each study contributed one estimate for each effect described above from Studies 2 to 4 (using the control condition in Study 4). I also examined the effects of appropriation ambiguity (ambiguous appropriation vs. overt cultural appropriation) in Black and White perceivers, for two effects in each of four studies representing the simple effects in the 2 (participant race) × 2 (appropriation ambiguity) design.

Using Excel-based workbooks for meta-analysis by Suurmond et al. (2017), for each study, I computed effect sizes along with the overall meta-analytic effect size (Cohen’s *d*). Results are summarized in Table 6 (perceived harm) and Table 7 (attributions of intent). The ambiguous cultural appropriation effect size for perceived harm was medium, *d =* 0.55, 95% CI = [0.3, 0.8]; Black perceivers evaluated more intergroup harm than White perceivers when reading about ambiguous cases of cultural appropriation. The overt cultural appropriation effect size for perceived harm was small, *d =* 0.26, 95% CI = [0.04, 0.47]; Black perceivers evaluated more intergroup harm than White perceivers when reading about overt cases of cultural appropriation. Overt appropriation was evaluated as more harmful than ambiguous appropriation, though more so among White perceivers, *d* = 1.57, 95% CI = [0.78, 2.36], than Black perceivers, *d* = 0.71, 95% CI = [0.02, 1.40].

**Table 6.** Meta-Analysis Results for Perceived Harm Across Studies 1 to 4, by Effect.

Study	Effect size (*d*)
Black–White (ambiguous appropriation)	
1	0.6
2	0.59
3	0.27
4	0.82
**Combined**	**0.55**
Black–White (overt appropriation)	
1	0.48
2	0.007
3	0.26
**Combined**	**0.26**
Appropriation ambiguity effect for White perceivers	
1	1.82
2	2.13
3	0.82
**Combined**	**1.57**
Appropriation ambiguity effect for Black perceivers	
1	0.52
2	1.42
3	0.24
**Combined**	**0.71**

**Table 7.** Meta-Analysis Results for Perceived Intent Across Studies 1 to 4, by Effect.

Study	Effect size (*d*)
Black–White (ambiguous appropriation)	
2	0.59
3	0.27
4	0.82
**Combined**	**0.55**
Black–White (overt appropriation)	
2	0.007
3	0.26
**Combined**	**0.18**
Appropriation ambiguity effect for White perceivers	
2	2.13
3	0.82
**Combined**	**2.24**
Appropriation ambiguity effect for Black perceivers	
2	1.42
3	0.24
**Combined**	**1.12**

The intent as justification hypothesis (H2) effect was large when reading about ambiguous appropriation, *d* = 0.50, 95% CI = [0.3, 0.71]; White perceivers attributed more positive intent to targets than Black perceivers. However, when reading about overt appropriation, the effect size was small, *d* = 0.18, 95% CI = [−0.06, 0.43]. Finally, targets engaged in ambiguous appropriation were attributed with more positive intent than targets engaged in overt appropriation, though more so among White perceivers, *d* = 2.24, 95% CI = [0.63, 3.86], than Black perceivers, *d* = 1.12, 95% CI = [−0.63, 2.88].

## General Discussion

To what extent are people’s evaluations harm as it relates to cultural appropriation a manifestation of aversive racism? According to the aversive racism theory of cultural appropriation, when situations are overt, and social norms and expectations are clear, dominant group members will be more likely to identify the harms enacted from acts of cultural appropriation. However, when situations are ambiguous, and the actor’s actions and behaviors can be “explained away” or justified, dominant group members will be less likely to “see” the harms associated with cultural appropriation, particularly because they will be more likely to emphasize the importance of actor’s intentions. Four experiments investigate the role of perceived actor intent in dominant and minoritized group members on the potential implications of harm enacted upon the source community as a result of culturally appropriative acts that are portrayed as ambiguous (vs. overt). Compared with minoritized group members, dominant group members were *less* likely to see the harm in ambiguous acts of cultural exploitation (confirming the *aversive racism theory of cultural appropriation*; H1), an effect mediated and explained by perceived actor intent (confirming *the intent hypothesis*; H2).

Study 1 provided initial evidence that White participants evaluated less harm than Black participants when making judgments about acts of ambiguous cultural exploitation. However, when reading about acts of overt appropriation, Black and White participants agreed on their evaluations harm. Study 2 replicated this effect and highlighted attributions of positive intent to the actor as an underlying mechanism for harm. In other words, compared with Black participants, White participants evaluated less harm in acts of ambiguous cultural appropriation because they attributed more positive intent to the target. Study 3 replicated this effect in a fully between subjects design using a new scenario of ambiguous cultural appropriation (art).

Study 4 sought to experimentally manipulate the intent of the target in a new scenario. When the target’s intent was portrayed as negative (and when intent was not explicitly mentioned), White participants evaluated less harm than Black participants. In contrast, Black and White participants only agreed on evaluations of harm when the target’s intent was clearly portrayed as positive. There are several reasons why Black and White participants may have evaluated similar levels of harm in the positive intention condition in an ambiguous case of cultural appropriation. Rationalizations that the actor “didn’t mean” to cause harm can allow individuals to deny the system of White supremacy and unconscious anti-Black sentiment by hyper focusing on intent over impact (Crandall & Eshleman, 2003; Gaertner & Dovidio, 1986). In contrast, Black participants may have been more hesitant to report perceived harm caused by a positively intentioned target for fears of being accused as oversensitive (Kaiser & Miller, 2001; West & Eaton, 2019), especially when the type of cultural appropriation is more ambiguous.

Even when Black people have a legitimate reason to suspect discrimination, they are more likely to be labeled as complainers when they confront discrimination (Kaiser & Miller, 2001). Minoritized groups are also more subject to hostile backlash and antagonistic reactions when they speak up and express agency (Czopp & Monteith, 2003; Moss-Racusin & Rudman, 2010; Moss-Racusin, Phelan, & Rudman, 2010; Rudman et al., 2012). Therefore, the risks of confronting ambiguous cultural appropriation may be particularly high for minoritized perceivers when perpetrator intentions are labeled as positive, as the burden of proof for confronting discriminatory action can become much greater than when labeled as negative (Haney-López, 2012). Focusing on actor intentions may have *actively silenced* marginalized voices seeking recognition and validation from injustice by motivating them to downplay their perceptions of harm (Durrheim, 2024; Teo, 2010), especially as they are less likely to be viewed as credible sources of their own lived experiences of discrimination (Czopp & Monteith, 2003; Diebels & Czopp, 2011; Garcia et al., 2005; Kaiser & Miller, 2001; Rasinski & Czopp, 2010; Wallace, Craig, & Wegner, 2024; Wallace et al., 2021). Nevertheless, it should be interpreted with caution to interpret null results, as more work is needed to uncover why group members did not differ with respect to perceived harm when target intent is positive in Study 4.

However, a key empirical finding from the current research is that Black participants evaluations of harm *differed* based on the intent of the target across the three conditions (positive intent, negative intent, control). However, White participants evaluated similar levels of harm in the positive intent condition and the control condition, and only evaluated a high degree of harm when intent was clearly negative. This finding suggests that White perceivers may be more likely to give actors the benefit of the doubt when evaluating the harm in ambiguous acts of cultural appropriation by assuming positive intent in neutral situations. These results are in line with previous work showing that White perceivers (but not Black perceivers) similarly evaluate a White actor who stereotypically “complimented” Black athlete as equally likable as when they withheld the compliment (Czopp, 2008).

Study 4 is the first of its kind to investigate the downstream consequences of perceived harm on collective action, where prior studies in cultural appropriation have focused on cognitions rather than behavioral implications. Overall, greater perceived harm predicted greater collective action, an effect significant for both White and Black perceivers, although stronger for White perceivers. Moreover, in the target intent manipulation, when intent was *not* mentioned, Black and White participants were *equally* likely to engage in collective action to confront anti-Black discrimination. However, group differences emerged in both the positive intent condition *and* in the negative intent condition where White participants were *less* likely to engage in collective action compared with Black participants. Black participants may have detected greater injustice in the actions of the positively intended *and* the negatively intentioned chef that motivated them to engage in greater collective action (van Zomeren et al., 2004). In contrast, White participants may have evaluated such actions as more procedurally fair and more legitimate (Bettencourt et al., 2001; Ellemers et al., 1993) and thus were less likely to “put their money where their mouth is” by refusing to engage in collective action behaviors to mitigate the harm (van Zomeren et al., 2004). Findings are in line with aversive racism theory that suggests a disconnect between self-reports and behavioral indicators, as self-reports are more likely to be influenced by motivations to appear non-prejudiced (Dovidio et al., 2002; Greenwald & Banaji, 1995).

### Theoretical and Translational Implications

For decades, the perceived ethical status of cultural appropriation has long been debated in a variety of different disciplines, especially related to the perceived authorization of its use and the potential implications for causing harm (see Nguyen & Strohl, 2019; Scafidi, 2005; Young, 2005, 2020). This is the first psychological work to experimentally manipulate the *overtness* of an act of cultural appropriation, as well as the first to directly manipulate *intentionality* on behalf of the appropriator. Moreover, this work focuses on key outcomes of perceived harm and collective action. The current paper demonstrates the importance of investigating people’s evaluations of intergroup harm in acts of cultural appropriation, particularly when the group associated with the cultural product has been historically subject to racial oppression. Such findings have important implications for practice, policy, and research by providing critical insight into the cognitive and motivational factors that influence people’s evaluations of harm in acts of cultural appropriation, which can then provide a praxis for psychological intervention and a prevention of intergroup harm. In addition to these insights, the current research advances the psychology of racism, theories of intention and motivation, and work on intergroup relations.

#### Advancing the Psychology of Racism

The current research combines and expands classic theories (Allport, 1954; Blumer, 1958; Bobo, 1988; Sherif, 1958; Sherif & Sherif, 1953) and contemporary theories racism (Crandall & Eshleman, 2003; Gaertner & Dovidio, 1986, 2000; McConahay, 1986; Pettigrew & Meertens, 1995; Salter & Adams, 2016) by demonstrating how group members make sense of and respond to different types of outgroup cultural use in terms of evaluations of harm enacted toward the source community. This work is the first to experimentally show that dominant group members are less likely to identify the harm implicated by cultural appropriation in ambiguous situations, as such evaluations may be guided motivations to justify such actions by attributing positive intent to the actor (e.g., Gaertner & Dovidio, 1986, 2000). The current work suggests that cognitive factors associated with the target (i.e., intention) and the context (i.e., ambiguity of the act) can serve as a psychological justification for dominant group members to excuse and rationalize culturally exploitative behaviors (Crandall & Eshleman, 2003). Indeed, cultural appropriation is an important global issue that warrants further investigation (Scafidi, 2005), especially racial tensions have become increasingly hostile and complex (Bhattacharyya et al., 2016; Winant, 2020). In the current research, White participants were more likely to assume that the target had positive intent, even when there was no mention or indication of it (Studies 2 and 3). Furthermore, inducing a focus on positive intent (Study 4) made no difference in White participants’ evaluations of harm compared with the control condition, suggesting that it was already assumed, corroborating previous research on the assumed morality and innocence of whiteness (Phillips & Lowery, 2018).

To only declare the harm associated with cultural appropriation when the target expresses overtly negative intentions of bigotry suggests that these psychological justifications strategically afford the denial of the realities regarding racial oppression (Crandall & Eshleman, 2003; Knowles et al., 2014). Many scholars have also discussed how the system of racism is embedded in the structure of our everyday worlds (Salter et al., 2018). Such a denial ignores the key role that dominant group members have in creating, shaping, and maintaining structural worlds that promote race-based hierarchies through selected preferences, practices and actions (Bonilla-Silva, 2006; Feagin, 2020; Salter & Adams, 2016), and the many strategies they engage in to deny and distance themselves from ideas of racial privilege (Knowles et al., 2014).

Understanding the psychology of cultural appropriation sheds light on the shifting and contemporary nature of racism and its insidious ability to manifest in ambiguous ways (Gaertner & Dovidio, 1986, 2000). The current research suggests that dominant group members are more apt to locate the harm in acts of cultural appropriation when they can emphasize *individual blame* (vs. systematic; Rucker & Richeson, 2021). This work suggests that emphasizing the role of actor intention in acts of cultural appropriation can direct an actor in the service of continued racial domination by absolving the ingroup of interpersonal guilt and thus moral culpability (Branscombe & Miron, 2004). To focus on intent and the individual tendencies of the actor can reproduce racial domination by focusing attention *away* from the ways that individuals maintain racialized contexts through racialized ways of seeing, being, and acting in the world (Salter & Adams, 2016; Salter et al., 2018).

Moreover, creating false sense of intergroup harmony by prioritizing intent over impact can leave minoritized groups *more* vulnerable to exploitation by disarming suspicion and collective action (Dixon et al., 2010; Durrheim, 2024; Jackson, 2020; Sidanius et al., 1996), and allowing bad actors to take advantage of the broader system that only calls out acts when substantial damage has been done (Etoke, 2022; Feagin, 2020; Phillips & Lowery, 2018). Thus, interventions should be directed toward enhancing social consciousness in order to identify and dismantle the cultural-psychological structures that facilitate motivations to engage in culturally appropriative actions in the first place (Bonam et al., 2019; Mosley & Heiphetz, 2021). Future research should continue to probe the contexts in which Black perceivers truly see dominant group actors as an ally, as someone who does intend to help, and take them on good faith that they are trying to pay homage to their culture, leading to greater anticipated support, trust, and respect (Johnson et al., 2019; Johnson & Pietri, 2025, 2024; Pietri et al., 2018). Moreover, future research should continue to explore how acts of cultural appropriation become normalized through the frequent and public use of the commodified cultural product, where pervasive and normative acts are viewed as more ambiguous, and thus less harmful (Crandall et al., 2002; Woodard, 2014), such as in the wearing of a particular hairstyle, or the participation in a “trendy” style of dance.

Moreover, future research should continue to explore how minoritized groups are excluded from dominant group member’s moral circles, leading to a climate of silencing and lack of support through system legitimizing ideologies (Geiger & Swim, 2016; Mosley & Heiphetz, 2021) by automatically presuming innocence for dominant group members (Phillips & Lowery, 2015). Such moral exclusion can cause double victimization through the perpetuation of stigma (Major & O’Brien, 2005), accusations of complaining (Kaiser & Miller, 2001), and enhanced intergroup hostility for “unacknowledged cultural appreciation” (Garcia et al., 2010). If an individual does not see the harm associated with cultural appropriation, they will be less likely to support reparations (Kraus & Vinluan, 2023), and other policies that protect communities from harm. Future work should also examine ways to reduce intergroup harm through interpersonal confrontation (Czopp et al., 2006), and collective action by dominant allies, which can enhance intergroup trust, solidarity, and validation of injustice (Pietri et al., 2024).

#### Understanding the Role of Intent and Extending Theories of Motivation

Corroborating the findings of previous work in social cognition (Alicke et al., 2015; Cushman, 2008; Folger & Cropanzano, 2001), legal studies (Dripps, 2003; see also Ames & Fiske, 2015), and evaluation of prejudice and discrimination (Swim et al., 2003), the current research demonstrates the crucial role of perpetrator intent in portraying culturally appropriative actions as just, moral, and fair.

As aversive racism theory would suggest (Gaertner & Dovidio, 1986, 2000), evaluations of target’s intentions when involved in outgroup cultural use may not be completely positive or negative, but complex, ambivalent, and multifaceted, where there are a variety of competing motivations, perhaps some that lie outside of conscious awareness. Context and social norms may also alter the extent to which particular actor intentions may be more valorized than others (Crandall et al., 2002), such as when the actor engages in behaviors of compensatory egalitarianism out of performative allyship in order to portray a non-prejudiced self-image (Moss-Racusin, Phelan, & Rudman, 2010). Thus, future research should expand on simple valenced intentions as simply “positive” and “negative” to a more nuanced and complex understanding of the cognitive and emotional ambivalence associated with culturally appropriative actions, including direct and an indirect assessment of people’s attitudes, motivations, and behaviors (Greenwald & Banaji, 1995).

#### Understanding the Role of Cultural Appropriation in Intergroup Relations

While the current research focuses on people’s evaluations of intergroup harm, findings have important implications for understanding the extent to which *actual realities* of intergroup harm can occur as a result of cultural appropriation, such as the reinforcement of intergroup oppression through processes of stereotyping, prejudice, discrimination, and dehumanization (Matthes, 2019). Several theories in psychology would suggest that mere exposure to cultural appropriation may change the nature of horizontal group categorizations of “us” and them” into vertical positions of “superiority” and “inferiority,” that promote harmful assumptions that the subordinated source community as *nonnormative, other, or different* (Allport, 1954; Blumer, 1958; Bobo, 1988; Gaertner & Dovidio, 1986; Sidanius & Pratto, 1999; Turner et al., 1987). This juxtaposition is manifested via a cultural context of zero-sum competition, where the benefits to superior group occur through cultural degradation (Matthes, 2019; Sherif, 1958). Moreover, cultural appropriation can promote harmful ideologies that pathologize the outgroup’s cultural values, communication styles, and ways of being by people placing them in direct contrast to the normative White ways of being, thus positioning the source community as “the effect to be explained” (Feagin, 2020; Hegarty & Pratto, 2001). The current research shows how minoritized individuals are more apt to see the realities of this harm, regardless of the target’s expressed intent. Nevertheless, the scholarly literature suggests that even with the most positive intentions, goals to commemorate a source community can inadvertently dehumanize them and their cultural products into an exoticized fetish (Buescher & Ono, 1996; Keefer et al., 2017; Kurtiş et al., 2010).

In addition to these theoretical implications, this research has translational implications for individuals who have positively intentioned motivations to engage in outgroup cultural use by suggesting that a critical reflection of one’s own sociopolitical location, group identities, and individual and collective motivations is a necessary first step to preventing harm enacted toward the source communities. A failure to do so may lead the actor, and perceivers, to underscore the potential harm that can take place as a result of cultural appropriation (Matthes, 2019). Awareness and interventions aimed at counteracting intergroup harm is important. Thus, individuals should also put forth effort into learning about the history of the cultural product, its cultural significance to the source community, as well as the lived experiences of the community members to gain a more holistic sense of the potential negative and positive implications that outgroup actions can have (Mosley et al., 2020; Mosley & Solomon, 2023). This research offers new insight and understanding regarding positive intention to truly honor outgroups, where there is true, respectful cultural appreciation that involves a sense of intimacy, investment and trust on a collective level with the source community (Nguyen & Strohl, 2019), and how motivations for authentic relation play a crucial role in how the source community can mitigate potential harm as a result of their behaviors.

### Limitations and Future Directions

While the current research has several theoretical and translational innovations regarding how people evaluate the harm of acts of culturally appropriation, there are confines that limit the generalization of this work that future research can address. One limitation of this work is the nature of the scenarios, as one cannot be sure how findings generalize across different acts of cultural appropriation that range in conceptual nature through different ontological manifestations (Young, 2005, 2010), ranging from more concrete, physical cultural products (*object appropriation*, such as hair, skin tone, or cuisine) to cultural products that are more abstract and conceptual in nature where the “cultural product” is the *perceived* subjective experience of a source community (*subject appropriation*, such as in literature, art, or cultural parties). While the current research explores subject appropriation (Studies 1–3) *and* object appropriation (Study 4), future research should directly compare the different forms of cultural appropriation.

For example, the appropriation of tangible and material cultural products (i.e., object appropriation) may be *perceived* as more harmful than abstract and intellectual products (e.g., subject appropriation, motif appropriation). Subject appropriation may facilitate the greatest perceptual differences because it refers to a symbolic construct that is completely abstract and immaterial in nature (Young, 2005, 2010). Furthermore, subject appropriation may lead to the greatest *actual* intergroup harm by influencing cultural learning process that perpetuate stereotypes about minoritized groups due to the performed or narrated subjectivity of a collective outgroup (Bandura & Walters, 1977). In such instances, the target takes on epistemological authority and assumes an existential way of being about the subjective experience of an outgroup source community through narration or performance (Buescher & Ono, 1996).

Such moral centering prioritizes and privileging the psychology, experiences, and therefore, the humanity of the dominant actor engaged in cultural appropriation *over and above* the minoritized source community (Ledgerwood et al., 2024; Starck et al., 2024; Torrez et al., 2024).

Moreover, focusing on actor intentions can contribute to an atmosphere of ontological violence allowing dominant group members to decontextualize and abstract the *behavior* of outgroup cultural use from *broader structure* of colonial racial violence (Adams et al., 2017; Phillips & Lowery, 2018). Thus, future research should examine whether hyperfocusing on positive actor intentions contributes to racial resentment and backlash at minoritized groups for being “ungrateful” that they are being “honored” in the first place, dismissing the possibility the said “honor” may be patronizing, paternalistic, or uncalled for (Wang et al., 2015, 2019).

Another limitation to the generalizability of this work is the nature of the participants and the source community. As with most work on racism (Sears et al., 1999), the current research specifically examines cultural appropriation in the context of Black-White relations in the United States. This choice was intentional because Black people have been historically subject to racial violence and terror, including chattel slavery, Jim Crow Laws, and pervasive publicized lynchings, where their very lives were subject to capitalistic exploitation, commodification, and dehumanization (Etoke, 2022; Hartman, 2022; Woodard, 2014). Thus, an empirical investigation of cultural appropriation in the United States, and its assessment of enacted harm, must fundamentally include the marginalized voices who have been highly subjected to overt and ambiguous forms of racial violence in the United States (Etoke, 2022; Hartman, 2022).

Furthermore, much scholarship has highlighted the invisible role of White supremacy in people’s denial acts of racism and cultural appropriation (Bonilla-Silva, 2006; Feagin, 2020; Knowles et al., 2014), and White people having the most power to dismantle systems of inequality enact positive social change (Knowles et al., 2014; Sidanius & Pratto, 1999). A major strength of this work is that it samples both individuals who are racialized as White and those who are racialized as Black, which contributes to the diversity of psychological science given Black participants’ scarcity of their inclusion (Roberts et al., 2020). However, future research is needed to investigate the extent to which the current findings generalize to other minoritized groups, as there are similarities in the nature of racial oppression (Cortland et al., 2017).

Moreover, this work is limited in that it does not take into consideration how the intersection of social identities such as race, gender, class, and sexuality influence the complex nature of harm associated with acts of cultural appropriation. Future research must take an intersectional lens to better understand how people with multiple subordinated social identities are potentially impacted by acts of cultural appropriation (Purdie-Vaughns & Eibach, 2008). For example, recent work has discussed how cultural appropriation of Native American dance can lead to sexual violence toward indigenous and Native women (McShane, 2023). International cultural studies have discussed how the outgroup cultural use of the artform of Japanese anime has the potential to distort racialized gender attitudes about Japanese women who are portrayed as characters by calling attention to their sexualized bodies and reinforcing objectification (Mertens, 2023). Furthermore, social psychology has discussed that the connection between a societal pre-occupation with Black women’s aesthetics and physicality (e.g., hair weaves, big butts) through cultural consumption can lead to objectification and animalistic dehumanization of Black women (Mosley, Bharj, & Biernat, 2023). Indeed, future research should explore how cultural appropriation can promote unique racialized gender stereotypes as they relate to intersecting hegemonic ideologies of androcentrism, ethnocentrism, and heterocentrism (Purdie-Vaughns & Eibach, 2008).

The current research also has important implications for understanding stigma internalization and appropriated racial oppression and is of relevance to clinicians, counselors, and mental health practitioners. For minoritized group members, the appropriated portrayal of their culture can be particularly stigmatizing because it purports ideologies that “no history has taken place” (Mills, 2019, p. 31), that they are incapable of culture and distinction (Haslam & Loughnan, 2014), or that minoritized individuals have limited and restricted ways of being in the world (Fryberg & Stephens, 2010). Some acts of cultural appropriation may be experienced as particularly traumatic and harm psychological well-being due to enhanced threats to value, continuity, and meaning (Etoke, 2022; Jackson, 2020; Kashima, 2010; Lenard & Balint, 2020). Cultural movements such as Afrofuturism depict the pressing need for minoritized groups to have sovereignty over their representations and to transcend beyond systems of oppression that provide them with a sense of meaning and identity continuity (Barber et al., 2015; Eseonu & Duggan, 2022; Eshun, 2003; Jackson, 2020; McAdams, 2001; Yaszek, 2006). Future research should explore whether the subtlety of harm in acts of ambiguous cultural appropriation can lead to even more negative emotional, physiological, and behavioral responses among minoritized individuals than overt ones (Mendes et al., 2008).

## Conclusion

Many scholars have discussed how cultural appropriation can serve as a form of epistemic violence that subjugates already oppressed groups into further marginalization by denying the intellectual sovereignty, autonomy, and humanity of the source community members (Etoke, 2022; Matthes, 2019; Rogers, 2006). The current research shows how focusing on intent can be harmful by denying the realities and lived experiences of marginalized group members who have been historically subject to racial injustice (Eberhardt, 2019; Hartman, 2022; Roberts & Rizzo, 2021). If there is greater perceptual emphasis placed on the individual motivations of the actor (e.g., “he didn’t mean to cause harm,” “she had positive intentions”), attention and resources are diverted away from the impact, thus suppressing potential change toward more equal social relations (Dixon et al., 2010, Durrheim, 2024).

The goal of this work is not to invalidate the role of positive target intention, or downplay a target’s autonomy and will to celebrate the outgroup culture, but to highlight the motivational and sociocognitive nature that influences people’s judgments about intergroup harm. To ignore the harm that takes place, or to discount it as a result of perceived intention, is to cause additional harm to the source community denying their subjectivity and experiences of collective trauma. Furthermore, to the extent that harm could only be recognized by White perceivers when intent is undeniably negative falsely equates that the negative impact of cultural appropriation to a select few individuals (i.e., cultural appropriation is only perpetrated by “a few bad apples”), rather than a historical system of unequal intergroup relations (Adams et al., 2008). Therefore, no changes are made to improve the system, or the lives of those who are inadvertently affected by it (Dixon et al., 2010; Feagin, 2020; Rucker & Richeson, 2021).

## Supplemental Material

sj-docx-1-psp-10.1177_01461672241292427 – Supplemental material for The Aversive Racism Theory of Cultural Appropriation: Attributions of Target Intent Suppresses Evaluations of Intergroup Harm Supplemental material, sj-docx-1-psp-10.1177_01461672241292427 for The Aversive Racism Theory of Cultural Appropriation: Attributions of Target Intent Suppresses Evaluations of Intergroup Harm by Ariel J. Mosley in Personality and Social Psychology Bulletin

## Notes

1. As exploratory measures, this work also assessed perceived anti-Black discrimination and collective action. Means on this measure are included in the supplement.
