# Supplementary material for: The Aversive Racism Theory of Cultural Appropriation: Attributions of Target Intent Suppresses Evaluations of Intergroup Harm
Source: Pers Soc Psychol Bull. 2024 Dec 13;52(4):964–91. doi: 10.1177/01461672241292427 (PMC12949756; doi:10.1177/01461672241292427)
Supplement: sj-docx-1-psp-10.1177_01461672241292427 – Supplemental material for The Aversive Racism Theory of Cultural Appropriation: Attributions of Target Intent Suppresses Evaluations of Intergroup Harm [file sj-docx-1-psp-10.1177_01461672241292427.docx]

**Supplemental Materials**

**Appendix A. Stimuli for Studies 1 & 2**

**Appendix B. Stimuli for Study 2**

**Appendix C. Stimuli for Study 3**

**Appendix D. Dependent Measures Across Studies 1-4**

**Appendix E. Dependent Measures Not Included in Analysis Study 3**

**Appendix F. Means and Standard Deviations for Dependent Measures Not Included in Analysis Study 3**

**Appendix A. Stimuli for Studies 1 & 2**

**Ambiguous Cultural Appropriation Condition (Mosley & Biernat, 2021)**

**Author of Literature**

Kathryn Stockett is a Caucasian-American author. She is known for her 2009 debut novel, the Help, which is about African-American maids working in White households in Jackson, Mississippi, during the 1960’s. The book focuses on the friendships of three woman: a young White woman, skeeter, who aspires to be a writer, and two African-American maids, Aibileen and Minny. Critics have argued that Stockett’s depiction of Black characters are misinformed and stereotypical, and some have called the author out for cultural appropriation.


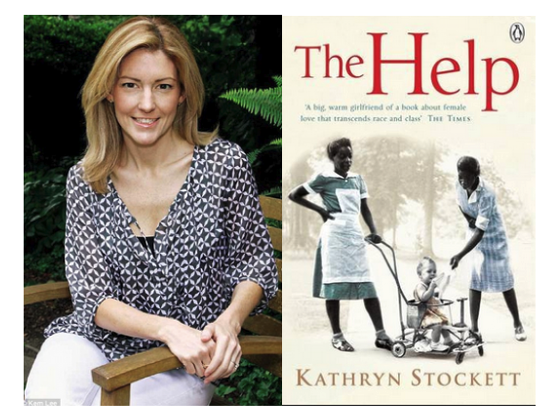
**What do YOU think?**

**Overt Cultural Appropriation Condition (Mosley & Biernat, 2021)**

**College Fraternity Parties**

Administrators at the University of California, San Diego are condemning a weekend “ghetto-themed” party thrown off-campus by a White fraternity to commemorate Black History Month. The “Compton Cookout” event urged all participants to wear chains, don cheap clothes, and speak very loudly, The San Diego Union-Tribune reported.

Female participants were encouraged to be ‘ghetto chicks.’ The invitation read, “For those of you who are unfamiliar with ghetto chicks – Ghetto chicks usually have gold teeth, start fights and drama, and wear cheap clothes…,” the Union-Tribune reported. Administrators are calling out the fraternity for cultural appropriation.


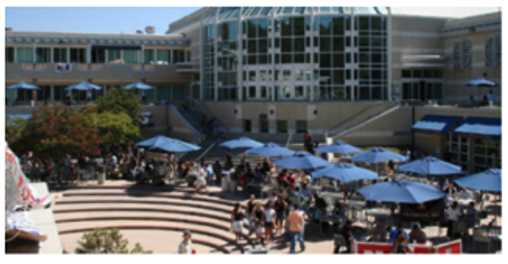
**What do YOU think?**

**Appendix B. Stimuli for Study 3 (Mosley & Biernat, 2021)**

**Ambiguous Cultural Appropriation Condition (Mosley & Biernat, 2021)**

**Art Exhibits**

Last year, the Contemporary Art Museum (CAM) in St. Louis debuted a solo exhibition by New York-based artist, Kelley Walker. The exhibition, titled “Direct Drive,” Walker solo in the United States, has drawn intense criticism as well as protests over his depiction of both Black women and of history. Immediately after the exhibition opened, the St. Louis Post-Dispatch reported that critics called for the “racially charged work to be removed and for the museum’s chief curator to step down.” Kelly Walker is a Caucasian-American artist whose latest project is the center of recent controversy.

The debate centers on two series featured in the exhibition, Black Start Press and schema; Aquafresh plus Crest with Whitening Expressions, both which data from the early 2000s and utilize appropriated photographs reprinted on canvas, covered with either toothpaste or chocolate. In the Black Star Press, Walker used iconic photographs from the 1963 civic rights movement, rotated the photographs each by 90 degrees, and covered them with melted dark and white chocolate. In “Schema,” Walker used enlarged covers of the now-defunct ING, a lad-mag-style publication geared towards Black men, featuring photographs of musicians Trina and Kelis, among others. Those images have been smeared with pastel-hued toothpaste. Now people are calling the artist out for “cultural appropriation.”

**What do YOU think?**

**
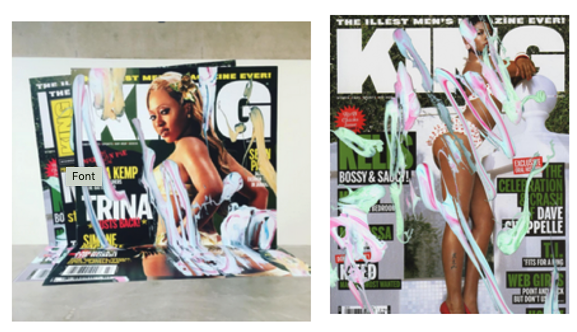
**

**Overt Cultural Appropriation Condition (Mosley & Biernat, 2021)**

**College Fraternity Parties**

Administrators at the University of California, San Diego are condemning a weekend “ghetto-themed” party thrown off-campus by a White fraternity to commemorate Black History Month. The “Compton Cookout” event urged all participants to wear chains, don cheap clothes, and speak very loudly, The San Diego Union-Tribune reported.

Female participants were encouraged to be ‘ghetto chicks.’ The invitation read, “For those of you who are unfamiliar with ghetto chicks – Ghetto chicks usually have gold teeth, start fights and drama, and wear cheap clothes…,” the Union-Tribune reported. Administrators are calling out the fraternity for cultural appropriation.


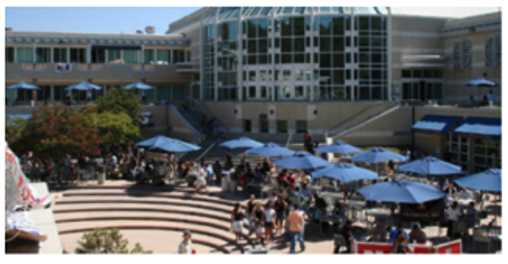
**What do YOU think?**

**Appendix C. Stimuli for Study 4 (Mosley & Biernat, 2021)**

**
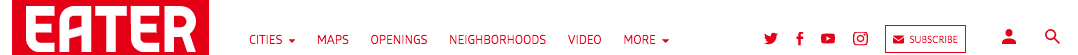
**


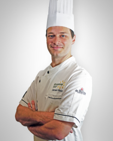


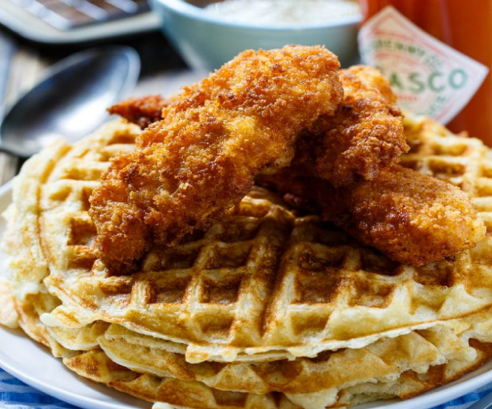
A trendy new restaurant, Nick’s Soul Food Renaissance is already being acclaimed for its popular and unique spin on cuisine such as deep fried chicken, hushpuppies, collard greens, chitlins, and black-eyed peas.

San Francisco chef Jamal Jefferson / Nick Miller, renowned chef to celebrities, who also [earned a Michelin star](https://sf.eater.com/maps/san-francisco-bay-area-michelin-restaurants-stars-2018) for his second restaurant [Octavia](https://sf.eater.com/venue/18864/octavia)nd, just opened up a new American soul food restaurant in downtown LA that boasts of all locally sourced ingredients. He's considered one of America's premier experts on soul food, and has grabbed quite a bit of media attention for his distinctive dishes.


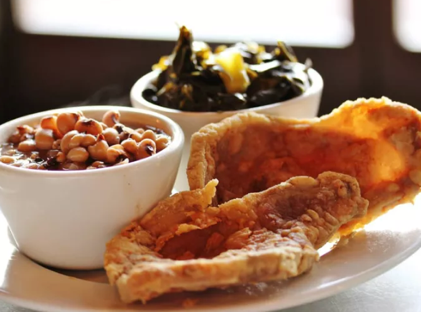
 Foodies are flocking to get a reservation at this popular restaurant, named the neighborhoods new “Up and Coming Place to Eat in LA.” The restaurant covers 4,200 square feet with an open kitchen, live-fire cooking, and room for outdoor dining. There is a full bar, lunch, brunch, and dinner menus, with occasional live music.


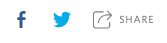


**Nick’s Soul Food Renaissance**

**“Specializing in Home Style Cooking”**

Specials Menu

**Nick’s Deep-Fried Chicken and Waffles Special…………………………….34**

**Served with brown gravy and Nick’s famous spices.**

**Country-Style Ribs with hushpuppies………………………………………………....32**

**Served with Rice, mustard collard greens**

**Famous Fried Chitlins and Black-Eyed Peas. ………………………………….…....29**

**Pig Intestines and black-eyed peas served with coleslaw**

**Crawfish Jambalaya………………………………………………………………..…....25**

Andouille smoked sausage, seasonal vegetables, and fresh seafood.

**Beef Oxtail…………………………………………………………………………..…....22**

**Served with fried green tomatoes, rice, gravy**


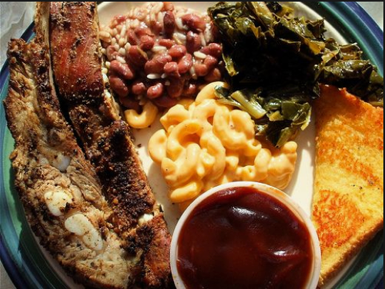

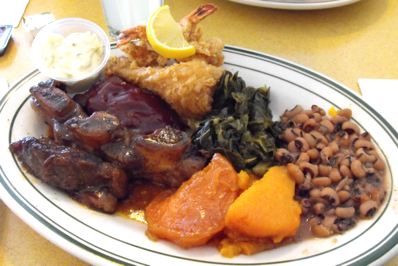


Positive Intent Condition

*
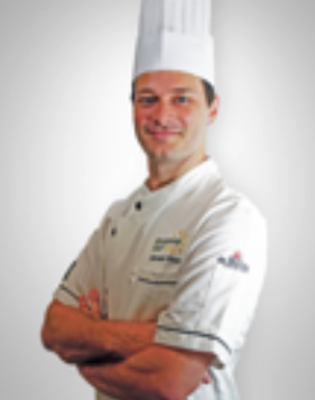
***When interviewed about his restaurant, Chef Miller responded,**

*“In my craft, I only have the* ***most positive intentions and honorable*** ***motivations*** *to honor Blacks and their culture, as society does not take them seriously enough. In the curation of my cuisine,* ***I only meant to celebrate soul food.*** *Throughout my career, I only have intended to create good for the Black community, with the ultimate goal of paying homage to Black culture.”*

Negative Intent Condition

*
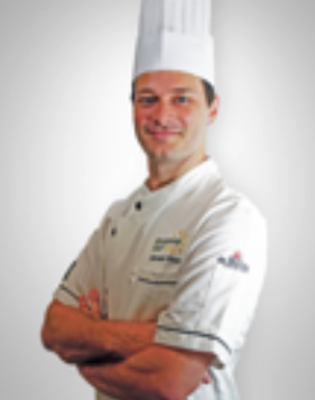
***When interviewed about his restaurant, Chef Miller responded,**

*“In my craft, I tend to have* ***aggressively competitive*** ***intentions*** *and* ***less than honorable motivations****. Many Blacks take themselves and their culture too seriously. In the curation of my cuisine,* ***I have intentionally tried to show how my food is superior to other soul food chefs****. Throughout my career, I only intended to the take advantage of every opportunity, with the ultimate goal of dominating the soul food industry.”*

Control Intent Condition

*
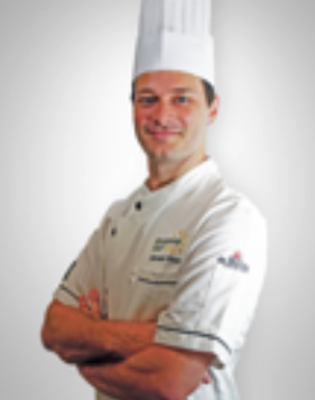
***When interviewed about his restaurant, Chef Miller responded,**

*“In my craft, I only use fresh, local ingredients and the finest cooking practices. Dishes that contain every possible flavor, sweet and sour, bitter, and fresh. Throughout my career, I have served the most delicious and popular culinary creations. Ingredients get a refined style. As a chef, I add my personal touch with a predilection for fresh tones, acids, and spices. Recognizable flavors are brought in an unexpected way.”*

**Appendix D. Dependent Measures Across Studies 1-4**

**Perceived extent of harm the target’s actions caused to Black culture** (1 = *Strongly Disagree* to 7 = *Strongly Agree;* Mosley & Biernat, 2021; Studies 1 - 3):

1. This person’s actions harm Black culture.
2. This person’s actions weaken Black culture.
3. This person’s actions demean Black culture.

**Perceived positive intentionality of the actor** (1 = *Strongly Disagree* to 7 =*Strongly Agree;* Mosley & Biernat, 2021; Study 2 only):

1. This person has good intentions.
2. This person has positive objectives.
3. This person is intentionally trying to create good.

**Manipulation Check of Appropriation Ambiguity** (1 = *Strongly Disagree* to 7 = *Strongly Agree;* Mosley et al., 2023):

1. This person is an overt act of cultural appropriation.

**Appendix E. Dependent Measures in Study 4**

**Perceptions of Discrimination [**Study 4; 1 = *Strongly Disagree* to 7 = *Strongly A*gree; Mosley & Brancombe, 2020; Schmitt, Branscombe, Kobrynowicz, & Owen, 2002]

1. Black people as a group have been unjustly victimized by society.
2. Black people as a group have been unfairly victimized because of their race.
3. Black people should work towards fighting anti-Black discrimination.
4. Black people often miss out on important opportunities because of their race.

**Collective Action** [Study 4; 1 = *Strongly Disagree* to 7 = *Strongly A*gree; Van Zomeren, Spears, Fischer & Leach (2004); Mosley & Brancombe, 2020]

1. I am motivated to confront future anti-Black discrimination.
2. I feel obligated to engage in collective action to fight anti-Black discrimination.
3. I would do something together with other people to call attention to anti-Blackness in society.
4. I would participate in a protest with other people to stop anti-Black discrimination.
5. I would support changes in policies that advocate for Black people’s rights.

**Appendix F. Means and Standard Deviations for Dependent Measures Not Included in Analysis Study 3**

| Table 3*.*  *Dependent measures by participant race and appropriation ambiguity, Study 3* | | | | |  |
| --- | --- | --- | --- | --- | --- |
|  | **Participant Race X Appropriation Ambiguity** | | | |  |
|  | **Ambiguous Appropriation** | | **Overt Appropriation** | | |
| **Measure** | **White Participants** | **Black**  **Participants** | **White**  **Participants** | **Black Participants** | |
|  | *M (SD)* | *M (SD)* | *M (SD)* | *M (SD)* | |
| Perceived Discrimination | 5.20 (1.63) | 5.95 (1.33) | 5.12 (1.99) | 6.04 (1.26) | |
| Collective Action | 4.50 (1.76) | 5.66 (1.27) | 4.60 (1.89) | 5.71 (1.06) | |
